# Chromatin Immunoprecipitation Reveals p53 Binding to G-Quadruplex DNA Sequences in Myeloid Leukemia Cell Lines

**DOI:** 10.1021/acsbiomedchemau.4c00124

**Published:** 2025-02-12

**Authors:** Libuše Kratochvilová, Alessandra Dinová, Natália Valková, Michaela Dobrovolná, Pedro A. Sánchez-Murcia, Václav Brázda

**Affiliations:** 1 Institute of Biophysics of the Czech Academy of Sciences, Královopolská 135, Brno 612 65, Czech Republic; 2 Department of Food Chemistry and Biotechnology, Faculty of Chemistry, Brno University of Technology, Purkyňova 118, Brno 612 00, Czech Republic; 3 Laboratory of Computer-Aided Molecular Design, Division of Medicinal Chemistry, Otto-Loewi Research Center, Neue Stiftingtalstr. 6/III, Graz A-8010, Austria; 4 BioTechMed-Graz, Mozartgasse 12/II, Graz A-8010, Austria

**Keywords:** G-quadruplexes, p53 proteins, p53 binding sites, transcription modulation, DNA structures

## Abstract

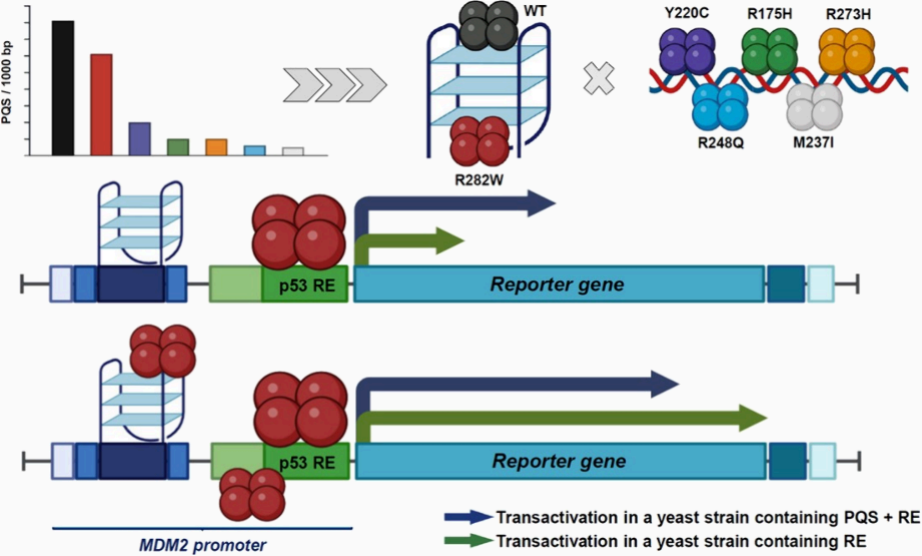

Clarifying functions of the p53 protein is a crucial aspect of cancer research. We analyzed the binding sites of p53 wild-type (WT) protein and its oncologically significant mutants and evaluated their transactivation properties using a functional yeast assay. Unlike the binding sites as determined in myeloid leukemia cell lines by chromatin immunoprecipitation of p53-R175H, p53-Y220C, p53-M237I, p53-R248Q, and p53-R273H mutants, the target sites of p53-WT and p53-R282W were significantly associated with putative G-quadruplex sequences (PQSs). Guanine-quadruplex (G-quadruplex or G4) formation in these sequences was evaluated by using a set of biophysical methods. G4s can modulate gene expression induced by p53. At low p53 expression level, PQS upstream of the p53-response element (RE) leads to greater gene expression induced by p53-R282W compared to that for the RE without PQS. Meanwhile, p53-WT protein expression is decreased by the PQS presence. At a high p53 expression level, the presence of PQS leads to a decreased expression of the reporter regardless of the distance and localization of the G4 from the RE.

## Introduction

The p53 protein is a transcription factor known as the guardian of the genome. It is a 44 kDa^1^ nuclear protein encoded by the *TP53* gene located on the short arm of chromosome 17.^2,3^ The wild-type p53 protein (p53-WT) plays key roles in many important biological processes by regulating the transcription of several target genes,^4^ thereby controlling cell proliferation, aging and DNA repair processes, as well as cell death in oncogenic cells.^5^ As a tumor suppressor, the p53 protein checks the correctness of the DNA sequence in cells during the cell cycle phase and, in case of damage, induces mechanisms leading to its repair. If DNA repair is ineffective, cells undergo programmed cell death, apoptosis, which prevents further transmission of damaged DNA during cell division and reduces the risk of developing cancer^2^.

*TP53* is the gene most frequently mutated in human cancers,^2,6^ but the processes by which its mutation or depletion leads to tumorigenesis still need to be better understood.^5^ Mutations of the p53 protein are especially widespread in high-prevalence cancers, including breast, colon, and lung cancer.^2^ Based on the p53 protein’s type of mutation, its function undergoes a complete loss of function (LOF), a partial loss of function (partial function [PF]),^7^ or a gain of new functions (gain of function [GOF]) that attenuate the development of cancer diseases.^6^ The different p53 protein mutations can lead to different types and strengths of GOF.^8^ The most common type (80–95% of cases) of p53 protein mutations consist of missense mutations.^4,6,9^ Missense mutations are reported to have functions different from those of p53-WT^5^ and are located predominantly in the DNA binding domain at residues R175, G245, R248, R249, R273, and R282.^9−11^ Mutations in codons R282, R175, G245, and R249 affect the structural integrity of p53 binding to DNA.^12^ The p53-R282 mutation has been more frequently detected in bone tumors, while the p53-R175 and p53-R248 mutations have been enriched in brain tumors.^8,9^ The p53-R282W mutant exhibits some extent of GOF and is strongly associated with the clinical prognosis of cancer patients.^9^ Loss of p53-WT function can be mediated by gene mutation leading to LOF (in some cases also GOF) of p53-WT or by overexpression of negative regulators of mouse double minute 2 homologue (MDM2) and its homologue murine double minute X (MDMX).^13,14^ The p53 protein is thus a promising potential target for the development of new anticancer drugs.^9,13^ Although newly developed MDM2 inhibitors^15,16^ and therapeutic agents targeting mutant p53 proteins restoring p53-WT function are currently in clinical trials and showing promising results, none of the agents have been approved by the United States Food and Drug Administration (FDA). Alternative strategies targeting mutant p53 proteins will be based on a better understanding of p53 biology, on further understanding of regulatory mechanisms, and on the effects of therapeutics targeting proteolysis for MDM2 degradation^13^.

The p53-WT protein specifically recognizes the consensus DNA sequence of two 5′-RRRC(A/T)(T/A)GYYY-3′ repeats (R = purines, Y = pyrimidines), which can be separated by a 0–13 bp spacer.^17^ The p53 protein binds a DNA target sequence as a tetramer.^18^ Although p53 binding to DNA is mediated mainly by the DNA binding domain, its C-terminal modification is necessary for efficient binding of the protein to DNA,^18,19^ with only the full-length protein able to recognize target sites efficiently in long DNA.^18,20,21^ In addition, p53 protein binds to non-B local DNA structures.^22^ It has been shown that p53 protein preferentially binds duplexes with mismatches, cruciform,^23^ bent DNA,^21^ structurally flexible chromatin,^24^ hemicatenated DNA^25^, DNA bulges, three-way or four-way junctions,^26^ telomere T-loops,^27^ and superhelical DNA.^28−30^ Structure-selective binding of p53-WT and p53-G245 proteins to intermolecular triplexes (dT)50(dA)50(dT)50 has also been reported.^18^ P53-WT binds triplex DNA at a level comparable to the hairpin structure with a significant contribution from the C-terminal domain, where the affinity of the full-length protein and the C-terminal domain alone to triplex DNA outweighs the affinity of the DNA binding domain alone.^31^ The wild-type protein p53-WT and some mutants, such as p53-G245S, p53-R248W, and p53-R273H, show *in vitro* an ability to interact with the secondary structures of guanine quadruplexes (G-quadruplexes or G4s).^32^ G4s are secondary noncanonical local structures of nucleic acids that are formed under physiological conditions from sequences rich in guanine bases.^33^ G4s are stabilized by sodium (Na^+^) or potassium cations (K^+^)^34^ and are found in important regulatory regions of the genome, such as gene promoters or telomeric DNA.^35^ Circular dichroism (CD) spectroscopy has demonstrated the ability of mutant p53 proteins to stabilize G4 assembly *in vitro.*(32) The p53 protein prefers telomeric G4 folded in the presence of K^+^ ions over G4 formed in the presence of Na^+^ ions.^36^ In addition to the effect of G4s on telomeres, G4s have been shown to affect transcription regulation,^37^ and the formation of multimolecular G4s in the context of chromatin could be a crucial structural feature for transcription regulation that needs further investigation.^38^ It also appears that there is an interplay between G4s and R-loops in the regulation of DNA repair, replication, and transcription in living cells.^39,40^ Regulation of transcription of target genes may be modulated not only by stabilizing G4 structures and affecting DNA–protein binding^41^ but also by some proteins; e.g., PCBP1 modulates transcription by recruiting the G4-specific helicase DHX9.^42^ Moreover, a presence of G4s has been demonstrated in up to 75% of binding regions in p53 mutants.^32^

A recently published study points out that p53-WT and p53-R282W proteins, in contrast to other p53 mutants, display large numbers of binding sites in the human genome.^43^ With a view to this study, we were interested in whether these binding sites contain sequences with the potential to form putative G-quadruplex sequences (PQSs) and how the presence of G4 structures found to be derived from promoter regions influences p53 protein binding and transcription induced *in vivo*. Therefore, while focusing on the p53-R282W mutant protein exhibiting GOF, we were the first to analyze data obtained from ChIP-seq analysis of the binding sites of p53 mutants and p53-WT for individual chromosomes. The analyzed binding sites were evaluated for the presence of PQSs by overlapping with the human genome hg38 and functionally classified within the overall genome. The representation of PQSs in the binding sites of individual p53 variants within the transcription start regions was evaluated, and the genes corresponding to the given regions were annotated. Through the thioflavin T (ThT) assay, the potential for G4 formation was confirmed in 13 more closely selected PQSs derived from protein coding genes. PQSs derived from the *MDM2* and *BBC3* (Bcl-2-binding component 3) genes were further subjected to CD spectroscopy analysis and cloned in the native configuration with the p53 response element (RE) into the yeast isogenic system to study the p53-WT and p53-R282W transactivation properties.

## Material and Methods

### Source and Editing of ChIP-Seq Data Sets

Chromatin immunoprecipitation sequencing (ChIP-seq) results were used to analyze sequences with the potential to form G4s located near the target sites of mutated p53 tumor suppressor proteins.^43^ Browser extensible data (BED) files of the hg38.p14 genome for the TP53-K562 cell line treated in two different environments (media containing dimethyl sulfoxide [DMSO] or daunorubicin) were downloaded from the ChIP-Atlas database. The analysis was performed for the data of all p53 protein variants (p53-WT plus six mutants: p53-R282W, p53-R248Q, p53-R273H, p53-R175H, p53-Y220C, and p53-M237I) used in the study. In the cases of p53-WT and p53-R282W proteins, which showed a similar PQS representation at the binding sites, additional analyses were performed to remove all overlapping sequences. Target sequences unique for the p53-R282W protein were obtained by subtracting those sequences interacting with the knockout p53 protein. The sequences for the p53-WT protein were obtained by subtracting the binding sequences of the p53 protein with the knockout state and/or the p53-R282W mutation. Sequences common to the binding of both proteins were obtained using the intersect function. Analyses were performed for K532 and MOLM13 cell lines, although for presentation in the main paper, mainly results for the K562 cell line are shown. Additional results for the MOLM13 cell line are shown in the Supporting Information. In addition to the p53 ChIP-seq results, we have also downloaded the experimental analyses of G4s by G-seq analyses of human genome^44^ and BG4-BG4-chip-seq data sets.^45^

### G4Hunter Analyses

PQSs in the hg38 genome were located using the G4Hunter software.^46^ The default parameters for G4Hunter analyses were used (G4Hunter score 1.2, windows 25 nucleotides). Those PQSs found were overlaid with adjusted ChIP-seq data files to find PQSs overlapping with DNA sequences precipitated by p53 and its mutant variations. The Python script “intersection.py” (Script S1) was used for the overlay (all Python scripts are available in the Supporting Information). The script produced pairs of files based on names and then traversed them looking for sequence intersections based on coordinates. For further analysis, the resulting CSV (comma-separated values) file containing the characteristics of PQSs and the respective p53 binding sites was divided into files based on the chromosomal localization of individual sequences.

### Randomization of Reference Human Genome

The sequence of the entire human genome was randomized 5× using Markov accounting relationships between individual bases in the sequence. Randomized genomes were analyzed for the presence of PQS by the G4Hunter software and overlaid with BED files using the script as in the case of the original sequence.

### Genome Annotation

PQS-containing targets were assigned to 2023 human genome hg38.p14 GENCODE annotations via the “intersection_annotation.py” script (Script S2). The script merged pairs of files based on their names and then searched for overlaps of annotated regions of the human genome and sequences containing target sites of p53 family proteins with PQS based on coordinates with respect to DNA strand orientation. Promoter regions (−1000 to +100 bp) from the start of transcription were annotated separately using the “intersection_promoter.py” script (Script S3), which works on a principle similar to that of the “intersection_annotation.py” script but uses only the position of the transcription start site instead of the coordinates of the annotated regions of the human genome and the adjacent promoter region of annotated transcripts with respect to DNA strand orientation.

### P53retriever

The binding sites of p53-WT or its mutant variants were analyzed for the presence of REs in the transcription origins (−1000 to +100 bp) by the p53retriever algorithm available in the R package.^47^ Sequences used for analysis were generated based on coordinates of the promoter regions of the “getfast” functions from the BEDTools toolkit.^48^ The analyzed sequences were assigned functional scores (grades) in the range of 1–5 based on the predicted transcriptional activation potential of the p53 protein, where the value 1 is assigned to those sequences with a probably nonfunctional RE and the value 5 is assigned to REs with predicted high transcriptional activity of the p53 protein.

### G-Quadruplex Formation Assays

#### ThT Assay

ThT is a fluorescent probe developed to identify amyloid fibrils;^49^ its ability to recognize G4 was subsequently confirmed.^50,51^ The interaction with G4 results in a restriction of the molecule’s rotation at the C–C bond between the benzothiazole and dimethylaniline parts, which induces a high fluorescence intensity.^52^ Stock oligonucleotides (Table 1) were dissolved in deionized water at a concentration of 100 μM. DNA samples were prepared from stock DNA at a concentration of 2 μM in 100 mM Tris-HCl (pH 7.4) or 100 mM Tris-HCl (pH 7.4) with the addition of 100 mM KCl to stabilize the G4 structure. Oligonucleotides were denatured at 95 °C for 5 min and slowly cooled to room temperature. An equimolar volume of 1 μM ThT was added to the oligonucleotide samples (resulting DNA/ThT concentration ratios were 0.1:0.5). Samples were measured in triplicate. Fluorescence intensity was measured in a 384-well Corning-brand microtiter plate on an Agilent BioTek Synergy H1 microplate reader. The fluorescence intensity of the samples was normalized to that of the ThT buffer alone, which also served as a negative control. The averages of determined *I*/*I*_0_ and sample standard deviation values were plotted in a graph (Figure 5).

**Table 1:** Oligonucleotide Sequences analyzed by PQS and Their G4Hunter Scores

**oligonucleotide**	**sequences**5′–3′	**RE–PQS distance [bp]**	**G4Hunter score [-]**
PCNA	CGGGTTCAGGAGTCAAAGAGGCGGGGA	8	1.240
GDF15	AGGGAGGGGTGGGTGAGGC	8	2.000
BBC3-01	ACTTGTCCGCGGCGGGCGGGCGGGG	overlap	1.240
BBC3-02	CGGGGCGGGGCGGGGCGGGGC	10	2.810
PIDD1	TGGGCGATGGCTGCAACGGTGGAGGGGC	21	1.346
INPP5B	GTGGCGCAGGGGCTGTTGGGAAATG	17	1.200
ASTN2	CGGGGCAAGGCGGCCCTGCAGGGGTGA	22	1.160
TRIM32	TGGGGAGGCGGGGCTCAGTGACGGACAGGGA	overlap	1.484
PURPL	TGGGGCAAGTGGGTGGAGCCATGAGGA	30	1.240
RPS19-01	TGGGCCCCGGGGGGCAGCGGCGGGGT	26	1.346
RPS19-02	CGGGGGGCAGCGGCGGGGTGCGTGGGGCGTCCGGA	32	1.657
CXCR2	AGTCCGTTGGCGGGGGCTGGGATG	overlap	1.250
MDM2	GGGCGGGATTGGGCCGGTTCAGTGGG	30	1.346

#### CD Spectroscopy

BBC3-01 and MDM2 stock oligonucleotides (Table 1) were dissolved in deionized water at a concentration of 100 μM. DNA samples with a concentration of 60 μM (converted to the total sequence length) were prepared from stock DNA in 10 mM Tris-HCl (pH 7.4) or 10 mM Tris-HCl (pH 7.4) with the addition of 100 mM KCl to stabilize the G4 structure. Oligonucleotides were denatured at 95 °C for 5 min and slowly cooled to room temperature. Spectra were measured on a Jasco 815 circular dichroism spectrometer in 1 cm tapered quartz cuvettes that were placed in a thermostatically controlled holder at 20 °C. Four scans of each sample were taken at a scan speed of 100 nm/min with data spacing of 0.5 nm in the wavelength range 200–330 nm and averaged, and the resulting spectra were smoothed using a Savitzky–Golay smoothing algorithm with a 15-point window. The CD signal was expressed as the difference in the molar absorption coefficient Δε of the left and right polarized light. CD measurements were also performed after 24 h.

### Luciferase Reporter Assay

Yeast strains were prepared by the Delitto Perfetto site-specific mutagenesis method.^53^*Saccharomyces cerevisiae* ISce-I endonuclease under the *GAL1* promoter (yLFM-ICORE), counter selectable *URA3*, and reporter *KanMX4* conferring kanamycin resistance yeast strains were modified to XV yeast chromosome upstream of the minimal yeast *pCYC1* promoter and the *LUC1* reporter gene derived from the firefly gene for luciferase expression.^54^ The iCORE sequence was replaced by sequences containing REs and PQSs with confirmed G4 formation. Sequences for expression of the p53 upregulated modulator of apoptosis (PUMA) proapoptotic protein from the *BBC3* gene and sequences derived from the MDM2 negative regulator of p53 were inserted in (Table 2). BBC3_RE contains the RE of p53 protein itself. BBC3 contains REs and PQSs of the *BBC3* gene derived in the native arrangement, including the overlap of RE and PQS. MDM2_RE contains the RE of the p53 protein itself, and MDM2 contains the RE and PQS of the *MDM2* gene derived in the native arrangement. Yeast strains were transformed with p53-WT and p53-R282W protein expression plasmids with the *GAL1,10* promoter for moderate to high protein expression and then selected. Transformants were resuspended in selective medium containing 0.016 and 1.000% supplemental galactose (GAL), which induced p53 protein expression. After 6 and 24 h (times 6:00 and 24:00), the established cultures were measured for OD_600_. Ten microliters of cell suspension was taken from each sample and lysed with an equimolar volume of 2× passive lysis buffer with constant shaking at room temperature for 15 min. Ten microliters of Bright Glo Luciferase Assay (luciferase substrate) was added to the lysed cells, and the bioluminescence value was immediately measured on an Agilent BioTek Synergy H1 microplate reader. The bioluminescence signal was normalized to the OD_600_ value (RLU) and recalculated against the normalized signal (RLU) of empty vectors (transformants containing appropriate selection markers without the expression of p53 proteins). Values exceeding 1.5 times the interquartile variance were removed from the resulting folder of empty values, and data were analyzed by ordinary two-way ANOVA with Dunnett’s multiple comparison test.

**Table 2:** RE and PQS Sequences Were Derived from Promoter Regions of Selected Genes Cloned into the Yeast Strain yLFM-ICORE

**yeast strain**	**sequences**5′-3′
BBC3_RE (PUMA)	CTGCAAGTCCTGACTTGTCC
BBC3	CTGCAAGTCCTG**ACTTGTCCGCGGCGGGCGGGCGGGG**
MDM2_RE	GGTCAAGTTCAGACACGTTC
MDM2	**GGGCGGGATTGGGCCGGTTCAGTGGG**CAGGTTGACTCAGCTT TTCCTCTTGAGCTGGTCAAGTTCAGACACGTTC

### Molecular Modeling of p53 Bound to MDM2 G-Quadruplex

The initial geometries for the complex between wild-type p53 (UniProt ID P04637, canonical sequence) and the GoF p53 variant R282W with the G-quadruplex of MDM2 (sequence 5′-GGGCGGGATTGGGCCGGTTCAGTGGG-3′) were generated with AlphaFold3 via the AlphaFold server (https://alphafoldserver.com^55^). Both DNA-binding domains in p53 (residues 98–292) and DNA partners were predicted with high confidence within the AlphaFold metrics. Important for the structure prediction of a parallel G-quadruplex was the inclusion of three potassium ions in the model. Otherwise, the AlphaFold3 prediction turned out to be a B-DNA with low confidence. The resulting p53/G4_MDM2_ complex was submitted to classical molecular dynamics (MD) simulations with explicit aqueous solvation. For p53, only the DNA-binding domain was included in each case (residues 98–292). The complexes were embedded into a box of OPC water molecules^56^ together with 16 Na+ ions to neutralize the solute. The atoms of the DNA G-quadruplexes were represented with the force field parameters BSC1,^57^ specially designed for nucleoside and nucleotides. The protein atoms were modeled via the Amber force field ff19SB^58^ and the counterions using the parameters of Li/Merz ion parameters.^59^ The solvated and neutralized system was minimized in three steps, where hydrogen atoms, solvent molecules, and the whole system were sequentially allowed to move during minimization. Then, the system was heated from 100 to 300 K in 200 ps at a constant volume (NVT ensemble) and using the Langevin thermostat (friction coefficient 1.0). During the heating process, the solute atoms were not allowed to move by imposing a harmonic constant of 200 kcal mol^–1^ Å^–2^. This constrains were slowly removed in five equilibration steps. In the last equilibration step, the system was simulated at a constant pressure (NPT ensemble). The systems were further simulated for 300 ns with a time step of 2 ps in GPU within the suite of software Amber20 using the program pmemd.cuda.^60−62^ The binding energies per residue were computed using MM-ISMSA^63^ by the analysis of 50 ns of 100 ns of the trajectory (100 snapshots).

## Results

### Presence of G-Quadruplex-Forming Sequences in p53-ChIPped Sequences

The important role of G4s in the DNA–protein interaction has been demonstrated in several studies.^64−68^ Some results also suggest the p53 protein’s ability to interact with G4.^69^ Therefore, we decided to analyze the presence of G4-forming sequences in the genome sequences immunoprecipitated by the p53 protein. We downloaded the ChIP-seq data sets of the Boettcher study,^43^ where ChIP of p53-DNA complexes was performed in cells with and without DNA damage induction treatment.

We first used the G4Hunter prediction tool^46^ to analyze immunoprecipitated sequences for the presence of the G4-forming sequences. As expected, the number of p53 protein binding sites increased in daunorubicin-treated cells compared to that in DMSO-treated cells. The greatest increase was demonstrated by the p53-WT (4.5×) and p53-R282W (2.7×) proteins followed by mutants p53-R248Q (1.9×), p53-Y220C (1.7×), p53-M237I (1.5×), and p53-R175H (1.3×). The R273H p53 mutant did not bind more after daunorubicin treatment (Tables S1 and S2, Figure S1). The tables and graph contain an overview of the number of binding sites for individual p53 and p53-WT mutants, including the results for our modified BED files, total lengths of binding sites, and representation of GC bases for individual chromosomes, and are divided according to the chemical with which the cells were treated.

**Figure 1:**
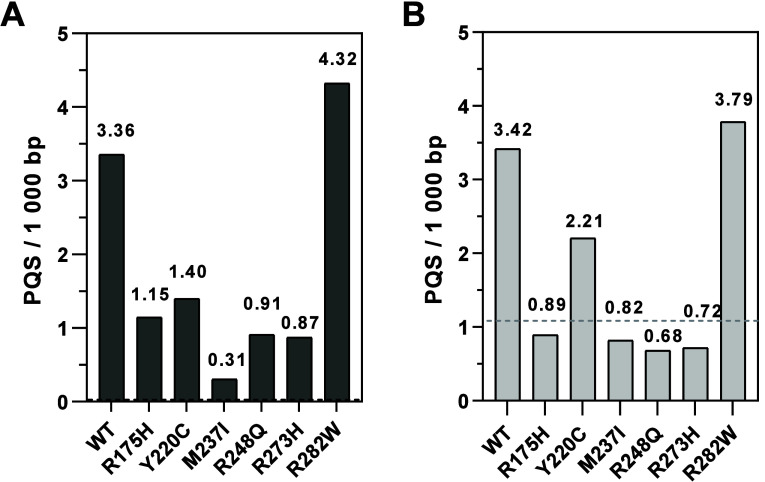
Frequency of PQS in binding sites of standard and mutant p53 protein in (A) DMSO and (B) daunorubicin medium. (A) In DMSO-treated cells, all binding sites of all analyzed p53 variants achieved higher frequency of PQS occurrence compared to the knockout condition, which in this case was equal to 0.00 (black broken line). The binding sites of p53-WT and mutant p53-R282W contained the highest numbers of PQSs. (B) In the daunorubicin environment, p53-WT and the p53-R282W mutant showed slightly lower abundance of PQSs within their target sites on DNA compared to the values for DMSO. For the other p53 variants, with the exception of p53-Y220C, the frequency of PQS was lower compared to the knockout condition with a value of 1.09 (gray broken line).

As the ChIP-seq results from the K562 cell line were annotated to hg38 assembly of the human genome, we have analyzed this assembly of human genome with the G4 prediction program G4Hunter^46^ using default parameters (G4Hunter score 1.2, window size of 25 bases). The overlap of G4-forming sequences demonstrated a high PQS frequency in the binding sites of p53-WT and p53-R282W proteins in both DMSO and daunorubicin (Figure 1). The PQS frequency in p53-WT binding sites was 3.36 in DMSO and 3.42 in daunorubicin. That was significantly higher compared to the values in knockout controls (0.00 in DMSO, 1.09 in daunorubicin). It is notable that the PQS frequency was higher for the p53-R282W protein (4.32 in DMSO, 3.79 in daunorubicin).

While the PQS frequencies in the binding sites of p53 proteins were significantly higher in DMSO compared to those in the knockout control (PQS frequency 0.00) for both p53-WT and all p53 mutants, PQS frequencies in the daunorubicin medium compared to those of the knockout control (1.09) were higher only for p53-WT, p53-Y220C, and p53-R282W proteins. The other proteins (p53-R175H, p53-M237I, p53-R248Q, and p53-R273H) showed PQS frequencies lower than those in the knockout state (Figure 1).

**Figure 2:**
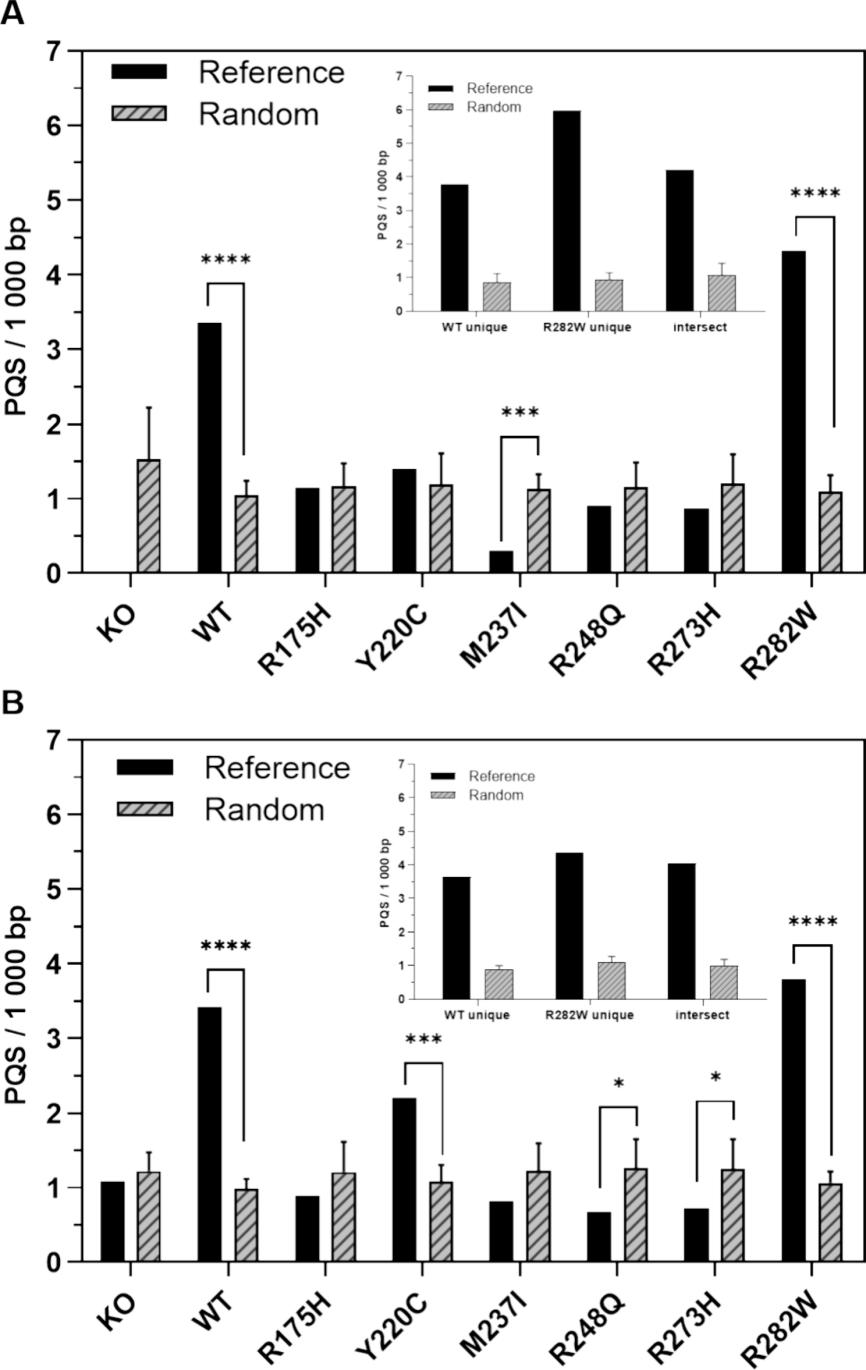
Comparison of PQS frequency in reference and randomized cell sequences of cells treated with (A) DMSO and (B) daunorubicin. (A) Reference sequences show statistically higher frequency of PQS occurrence in binding sites of p53-WT and p53-R282W and p53-Y220C proteins compared to randomized sequences. The protein binding sites of the remaining p53 protein variants showed a significant reduction in PQS frequency in the reference samples compared to the randomized sequences. (B) Compared to the randomized sequences, the reference sequences showed statistically higher frequency of PQS occurrence in the binding sites of p53-WT and p53-R282W proteins and lower frequency in p53-M237I. Randomized sequence values were determined as the mean of five analyses. The standard deviation is plotted. Statistical significance is marked at *p* ≤ 0.05 (*), *p* ≤ 0.001 (***), and *p* ≤ 0.0001 (****).

To evaluate the nonrandom presence of PQS in the immunoprecipitated sequences, we 5× randomized all tested fragments and performed G4Hunter analyses on the randomized sequences. While the PQS frequency in randomized sequences varied from 1.05 ± 0.19 to 1.53 ± 0.69 in DMSO and 0.98 ± 0.13 to 1.26 ± 0.39 in daunorubucine, the PQS frequencies in ChIP-seq sequences varied from 0.00 (knockout) to 4.32 (p53-R282W) in DMSO and 0.68 (p53-R248Q) to 3.79 (p53-R282W) in daunorubucine. We observed a significant change in PQS frequencies for p53-WT and p53-R282W proteins in both sample sets at *p* < 0.0001. A statistically significant higher frequency of PQS occurrence was shown also for proteins p53-Y220C, p53-R248Q, and p53-R273H in the DMSO environment and for the p53-M237I protein with daunorubicin treatment. Dependencies were also analyzed for sequences binding only p53-WT and p53-R282W proteins and their intersecting binding sites (Figure 2). The results of this analysis again showed a high frequency of PQS in cells treated with both DMSO and daunorubicin. In both media, a higher PQS frequency was found in the unique binding sites of the p53-R282W protein, and this was higher in DMSO (5.99) than in daunorubicin (4.36). For p53-WT, the frequency reached values of 3.77 in DMSO and 3.65 in daunorubicin, and the intersection of the binding sites of both proteins varied between the values for p53-WT and p53-R282W (4.21 in DMSO and 4.02 in daunorubicin), which again points to common binding properties of the two proteins.

### Colocalization of PQS and p53 Binding Sites with Functional Regions of the Genome

**Figure 3:**
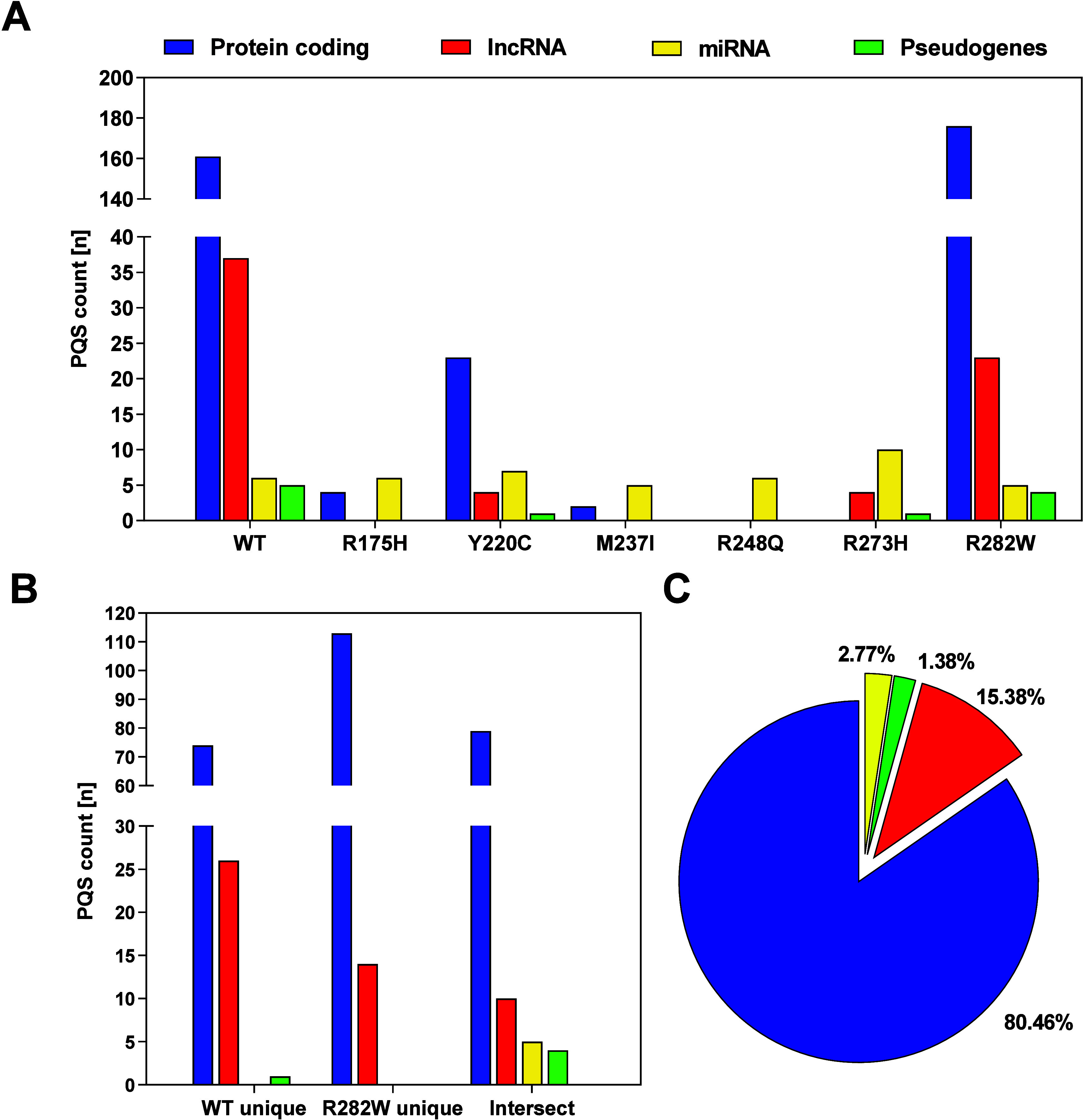
Functional classification of PQS within the human genome. (A) Analysis of the number of annotated PQS in the binding sites of individual p53 variants. (B) Analysis of the number of annotated PQS in binding sites for p53-WT, p53-R282W proteins only, and their common binding sites. (C) Percentage representation of PQS within protein coding regions, lncRNAs, miRNAs, and unprocessed and processed pseudogenes of all p53 variants.

To evaluate the association of PQS with the functional genome regions, we overlaid data sets with known annotations (Figure 3A). Of all of the sequences found (1857 sequences with PQS from a total of 2274 precipitated p53 binding sites), 490 sequences were overlaid with the already annotated sequences in the GENCODE database. The majority of sequences were annotated to protein-coding genes (80.50%) followed by long noncoding RNAs (lncRNAs, 15.40%), microRNAs (miRNAs, 2.77%), and pseudogenes (1.38%). The majority of protein-coding sequences were found in sequences precipitated by p53-WT and p53-R282W proteins (Figure 3B). PQSs in binding sites involving lncRNAs were present in all p53 variants except for the mutants p53-R248Q, p53-R175H, and p53-M237I. On the contrary, PQSs in miRNAs were present in the binding sites of all p53 variants except for the unique sequences of p53-WT and p53-R282W (Figure 3A,B).

**Figure 4:**
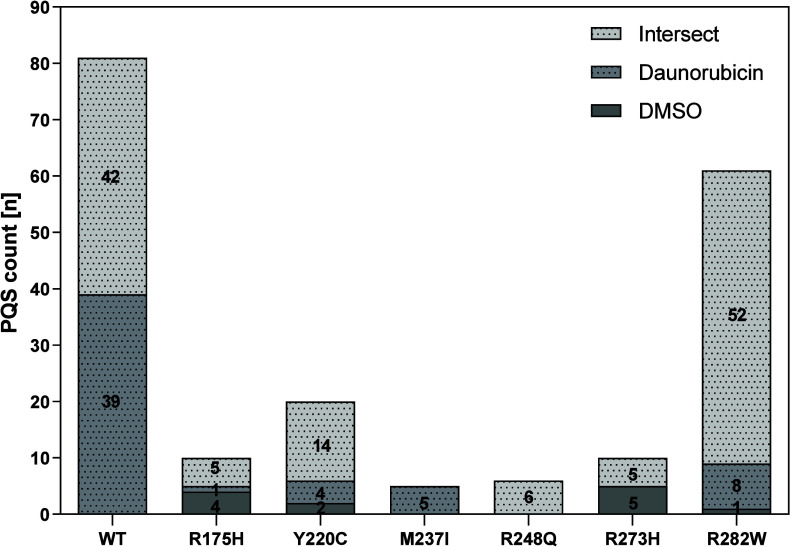
PQS representation in the binding sites of individual p53 variants in gene promoter regions. The highest numbers of PQSs were found in p53-WT^81^ and in the p53-R282W mutant.^61^ The fewest PQSs were found in p53-M237I^5^ and p53-R248Q.^6^ Data were analyzed for proteins in cells treated with DMSO alone or daunorubicin alone, or within their overlaps.

We furthermore analyzed PQS localization in the promoter regions (−1000 to +100 bp from the start of transcription) (Figure 4). A total of 115 different PQSs were found in the promoters of various genes bound by the p53-WT protein or its mutants (Figure S2). A large number of PQSs were bound by various p53 proteins, with the highest numbers of PQSs in binding sites found in p53-WT (total 81) and p53-R282W (total 61), which are the proteins that also showed the greatest PQS abundance in binding sites unique to each individual protein (without intersecting with other p53 proteins). Promoter regions were assigned to genes whose expression could be affected by the presence of G4 (Figure S2). Forty-eight different genes were found fulfilling the condition for the presence of at least one PQS in the promoter region. Most PQSs were found in the promoter region of the *PHLDA3* gene.^13^

In addition to PQS overlay analysis from *in silico* G4Hunter data, we used G-quadruplex coordinates from experimental analysis of G4s in the human genome by using G4-seq analysis. Since the original data were annotated for the human genome assembly 19, we first used the Lift Genome Annotations tool^70^ and then did an overlay of these data with the p53 ChIP-seq data set. Overlap of the experimental data set with the p53-ChIP data set was found for 1513 sequences (compared to 1857 overlapped sequences with PQS found with G4Hunter). Similarly, we found an overlap of the experimental G-quadruplex data set in promoter sequences with p53-ChIP data sets in about 30% of promoter sequences. Most importantly, both selected promoter sequences used for our experimental validation in the yeast isogenic system, the promoter sequences of MDM2 and BBRC3 genes, were presented also in experimental G4-data sets–G4-seq experimental data sets overlay with MDM2 promoter site and BG4-chip-seq data sets overlay with the BBC3 promoter site {Supplementary Table S6).

### Evidence for G-Quadruplex Formation in p53-Associated PQS

To study the transactivation potential of p53-WT and p53-R282W proteins in a chromatin context, we selected DNA sequences where REs and PQSs are located close in promoter sequences of protein-coding genes. We selected RE sequences showing high transactivation potential and PQSs with the highest probability for G4-formation as shown by the high G4Hunter score. The RE potential of the p53-WT and p53-R282W proteins’ binding sites was analyzed using the p53retriever program. Characterization of RE and PQS sequences for selected genes is shown in Table S4.

Of the 48 genes in which the presence of at least one PQS in the promoter region was demonstrated, 13 were selected where G4 formation was tested *in vitro* (Table 1). The selected PQSs were present not farther than 32 bp from the RE, with three of them (BBC3-01, TRIM32, and CXCR2) showing overlap with the RE. The p53retriever program assigned the REs of the respective genes to grades 2 and higher, and the G4Hunter scores ranged from 0.89 to 1.64 (Table 1).

**Figure 5:**
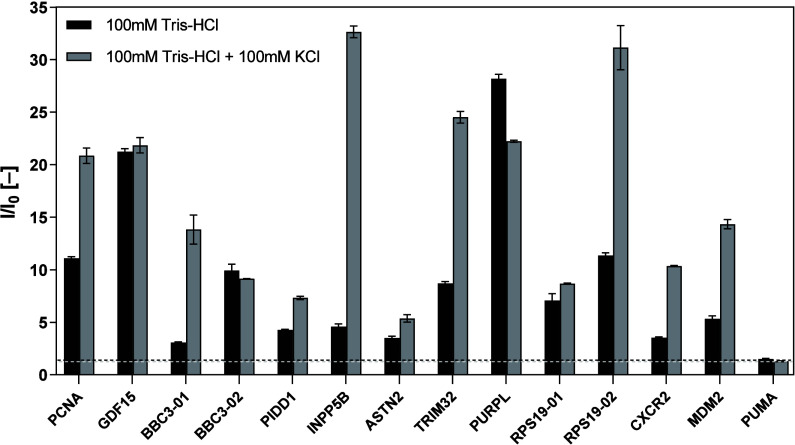
Results from biophysical characterization of selected oligonucleotides by the ThT assay. Oligonucleotides were hybridized in Tris-HCl (pH 7.4) or in the same buffer supplemented with 100 mM KCl. The fluorescence intensity (*I*) was normalized to the fluorescence intensity of ThT buffer alone (*I*_0_). Previously characterized PUMA oligonucleotide samples (RE protein p53 derived from the BBC3 gene; broken line) were used as a negative control.

The formation of G4 under physiological conditions from PQSs of 13 selected gene promoters was confirmed by the ThT assay (Figure 5). All analyzed samples reached *I*/*I*_0_ (intensity of fluorescence normalized to intensity of buffer itself with ThT) greater than the *I*/*I*_0_ PUMA sequence (negative control without G4 formation potential^71^), indicating at least partial G4 formation in the case of all the chosen PQSs. For all sequences except BBC3-02 and PURPL, a significant increase in *I*/*I*_0_ was observed in the G4 environment of stabilizing K^+^ ions. Samples of INPP5B (7.1-fold), BBC3-01 (4.5-fold), CXCR2 (2.9-fold), TRIM32 (2.8-fold), 12RPS19-01 (2.8-fold), and MDM2 (2.7-fold) showed the greatest fluorescence increases in the buffer with KCl compared with samples without KCl. In the remaining samples, the increases were less than 2-fold, indicating a lower level of stabilization or the formation of sufficiently stable G4 already in solution without stabilizing K^+^ ions. The slight decrease in fluorescence intensity of the BBC3-02 and PURPL samples with KCl compared to the sample in the buffer alone could be due to a change in the G4 conformation.^71^

Of the 13 G4-prone DNA analyzed, sequences derived from the *BBC3* and *MDM2* genes were selected for subsequent cloning into the yeast isogenic system to analyze the effect of G4 near the RE on transcription induced by p53-WT and p53-R282W proteins. The RE sequence of the *BBC3* gene had already been inserted into the PUMA yeast strain in the past, and an influence of G4 presence on the different level of transcription of p53-WT and its mutants or isoforms has been demonstrated, That system, however, was not derived from the native arrangement of the promoter region.^72,73^ The sequence derived from the promoter of the *MDM2* gene was chosen based on an earlier effort to understand the regulatory mechanisms of this important negative regulator of the protein p53 in the context of anticancer therapy. In addition, both *BBC3* and *MDM2* sequences showed a significant increase in *I*/*I*_0_ in the ThT assay.

**Figure 6:**
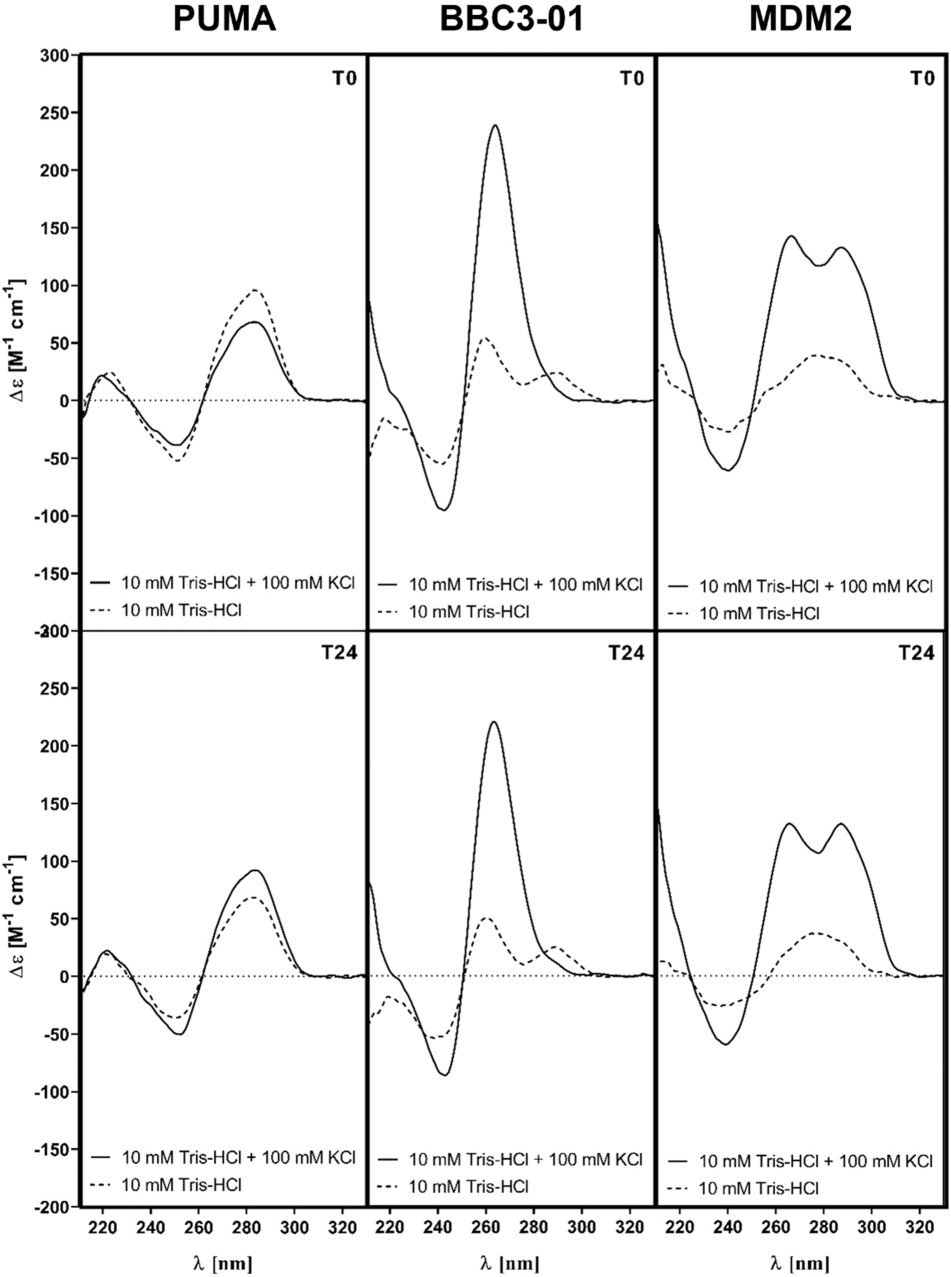
Results from the biophysical characterization of selected oligonucleotides by CD spectroscopy. T0 = time 00:00, T24 = time 24:00. The PUMA negative control does not form G4 even after 24 h. The spectra show significant positive peaks at wavelengths of 220 and 280 nm and a negative peak at 250 nm. In an environment of stabilizing potassium ions (solid line), BBC3-01 forms a parallel G4 structure whose conformation does not change even after 24 h. The spectra show significant positive peaks at wavelengths of 210 and ∼263 nm and a negative peak at 240 nm. In the environment without K+ ions (broken line), G4 formation was not observed even after 24 h. In an environment of stabilizing potassium ions (solid line), MDM2 forms a G4 hybrid structure whose conformation does not change even after 24 h. The spectra show significant positive peaks at wavelengths of 210, ∼263, and 295 nm. In the environment without K+ ions (broken line), G4 formation was not observed even after 24 h.

The formation and conformation of G4 from the MDM2 and BBC3-01 PQS sequences were further confirmed and characterized by CD spectroscopy. As a negative control, the oligonucleotide PUMA containing the RE sequence of the p53 protein derived from the promoter of the *BBC3* gene was used (Figure 6). Samples were hybridized in 10 mM Tris-HCl (pH 7.4) buffer or 10 mM Tris-HCl (pH 7.4) buffer supplemented with 100 mM KCl to stabilize G4 structures under physiological conditions and measured at times of 0:00 and 24:00. The measurement at 24:00 was used to test the change in conformation of G4 formed over time. Samples MDM2 and BBC3-01 clearly formed the G4 structure in the environment of K^+^ ions without a change in conformation after 24 h. The CD spectra of sample BBC3-01 acquire positive peaks at wavelengths of 210 and ∼263 nm and a negative peak at 240 nm, indicating the formation of a parallel G4 structure. In contrast, the CD spectra of the oligonucleotide MDM2 reach positive peaks at wavelengths of 210, ∼263, and 295 nm and indicate the formation of the G4 hybrid conformation. In contrast, the spectra of the PUMA oligonucleotide with the RE sequence of the *BBC3* gene did not show a presence of G4-specific peaks in any of the buffers. The spectra had positive peaks at wavelengths of 220 and 280 nm and a negative peak at approximately 250 nm, indicating the B-DNA structure.

### Transactivation Potential of p53-WT and p53-R282W Proteins

**Figure 7:**
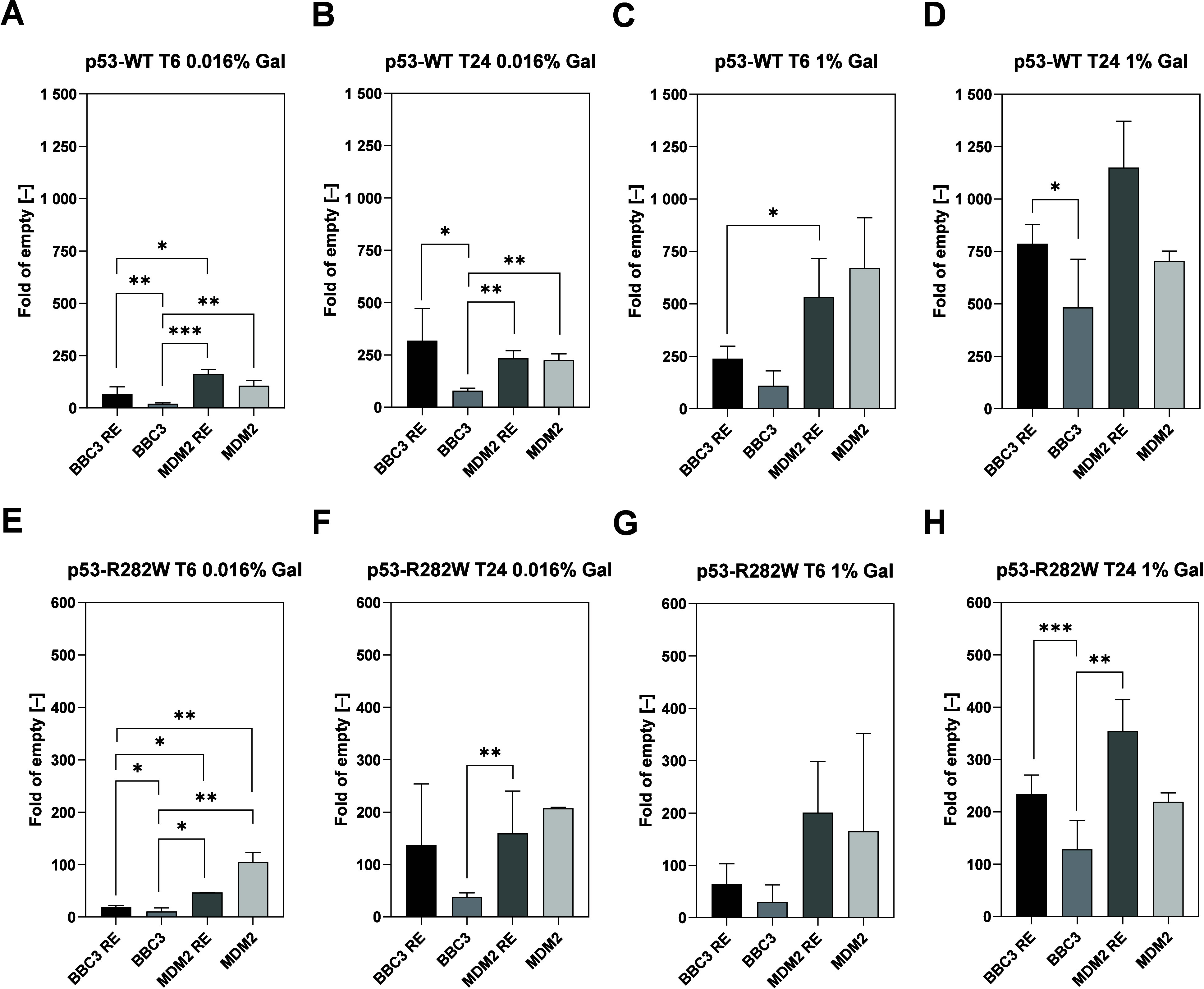
Modulation of gene expression induced by p53 family proteins. (A–D) Fold induction proteins p53-WT and (E–H) p53-R282W were determined after 6 h (A, C, E, G) and 24 h (B, D, F, H) of growth in 0.016% (A–B, E–F) and 1.000% (C–D, G–H) galactose. Bars plot the average fold induction and standard deviations of at least five independent replicates. Data were analyzed by ordinary two-way ANOVA with Dunnett’s multiple comparisons test. Statistical significance is indicated by asterisks: *p* < 0.05 (*), *p* < 0.01 (**), *p* < 0.001 (***), and *p* < 0.0001 (****). T6 = time 06:00, T24 = time 24:00.

The transactivation potential of p53-WT and p53-R282W proteins was analyzed in a one-hybrid yeast isogenic system. New *S. cerevisiae* yLFM-ICORE strains were prepared, where the ICORE cassette was replaced by either the p53 RE sequence alone or the RE sequence with the G4-forming sequence from the *BBC3* and *MDM2* promoters as determined by our bioinformatic analyses. Yeast strains were transformed by using plasmids with the *GAL1,10* promoter for inducible expression of p53-WT and p53-R282W proteins. Transcription induced by both proteins was studied using luciferase reporter assay at times 06:00 and 24:00 after establishing the transformants in media containing 0.016 and 1.000% galactose. The RE sequence was located upstream of the minimal yeast promoter and the *LUC1* gene. The level of transactivation (fold of empty) of p53 proteins was determined as the normalized bioluminescence signal, expressed as the relative light unit (RLU), against the RLU of empty vectors (without protein expression). Data were then analyzed by ordinary two-way ANOVA with Dunnett’s multiple comparisons test (Figure 7). The p53-WT protein (Figure 7A–D) showed a different level of transactivation within individual genes and a different level of protein expression at a given time. The presence of G4 downstream from the RE in the BBC3 strain resulted, in all measurements, in a statistically significant repression of reporter gene transcription (Figure 7A,B,D). In contrast to observations in the BBC3 strain, the presence of G4 in the MDM2 strain did not lead to a decrease in the level of transcription. MDM2_RE and MDM2 strains showed similar levels of reporter gene transcription that did not differ in a statistically significant manner. Compared to the BBC3_RE strain, however, the MDM2_RE strain showed a statistically significant increase in transcription induced by p53-WT at time 06:00 in the cases of both galactose concentrations (Figure 7A,C).

The p53-R282W protein showed approximately half of the level of transcriptional activation from what was seen for the p53-WT protein (Figure 7E–H). As in the case of p53-WT, the presence of G4 downstream from the p53 RE in the BBC3 strain led to a statistically significant decrease in the level of transcription (Figure 7E,H). Compared to p53-WT, a statistically significant greater transcription was noted in the MDM2 strain, even compared to both strains containing the RE. At a high level of protein expression, the p53-R282W protein showed a trend similar to that for p53-WT but without statistical significance (Figure 7D,H). Both proteins reached the highest level of transcription in the MDM2_RE strain and then in the BBC3_RE strain, while the presence of G4 downstream (BBC3) and even upstream (MDM2) led to a decrease in the transactivation of both proteins compared to control strains without G4.

### Interaction between p53 and MDM2 G-Quadruplex

**Figure 8:**
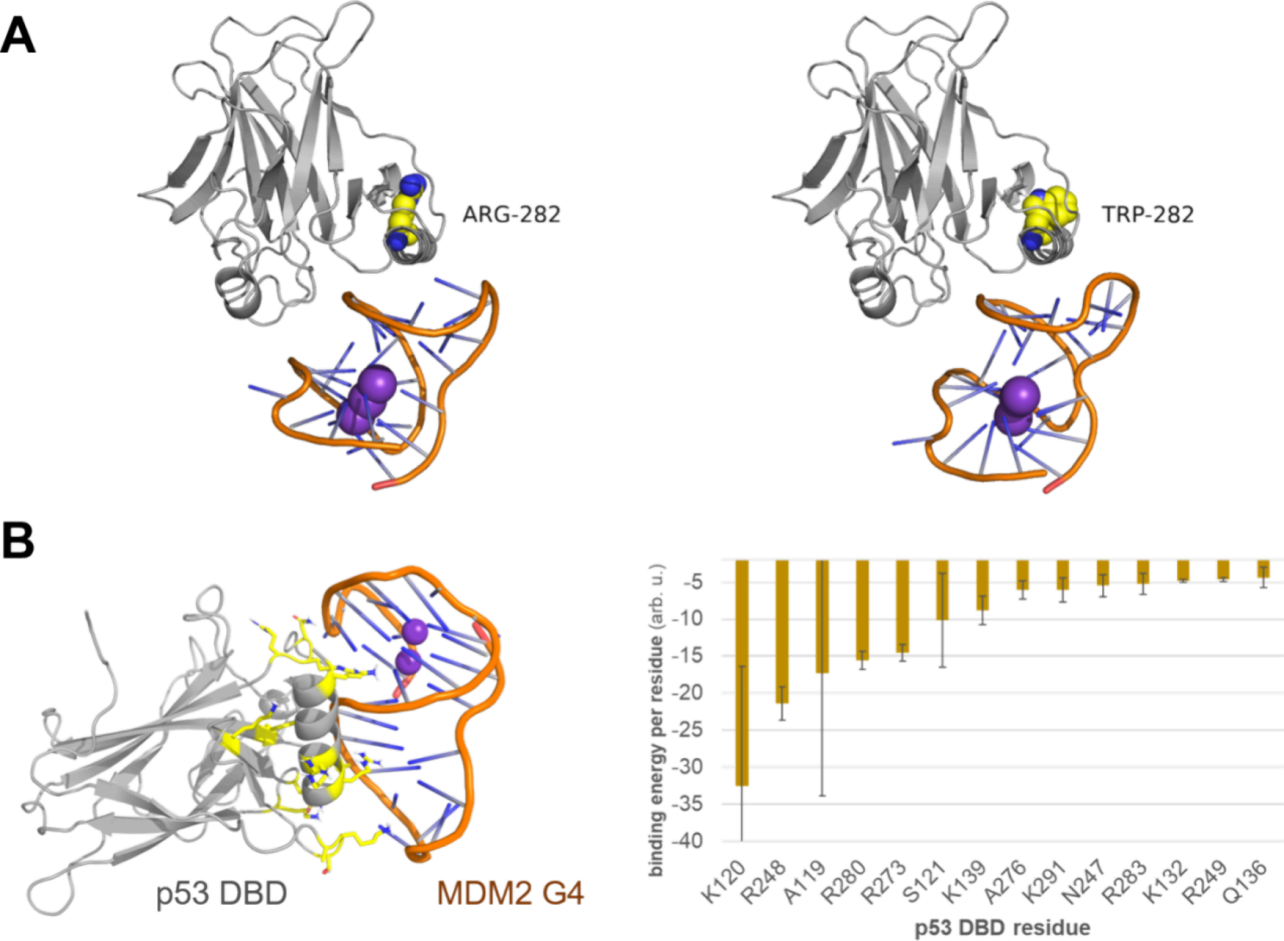
(A) AlphaFold3 models for wild-type and R282W p53 DNA-binding domains (gray cartoons) with MDM2 G4 (orange cartoons). Residue 282 is shown as spheres in both cases (C-atoms colored in yellow). (B) Relevant residues for the energetics of the binding between p53 and MDM2 G4 along the classical MD simulation. The key residues for the interactions between and p53 are calculated with MM-ISMSA.^63^

To elucidate how p53 may interact with MDM2 G4, we modeled wild-type p53 in complex with MDM2 G4 (Figure 8A). Starting from the AlphaFold3 model of the complex, we ran molecular dynamics (MD) simulations in water (Figure 8B). Since an important extension of p53 corresponds to intrinsically disordered regions, we included into our model the p53 residues 98–292, which correspond to the DNA-binding domain. The main interactions between p53 DBD and MDM2 G4 are mediated via the side chains of positively charged residues (i.e., K120, R248, R280, and R273).

## Discussion

The tumor suppressor p53 could boldly be identified as one of the most important transcription factors, as it plays crucial roles in DNA repair processes, apoptosis induction, cell cycle arrest, aging processes, immune response, and downstream regulation of target genes of the proapoptotic proteins Bcl-2 associated X, apoptosis regulator (BAX), PUMA, and p21.^74−79^ Compared to other tumor suppressors, the p53 protein is also exceptional in that it does not undergo deletions or other radical structural changes (as, for example, in such other tumor suppressors as retinoblastoma protein [RB], adenomatous polyposis coli [APC], or breast cancer 1 [BRCA1]) but mainly displays missense mutations in the central DNA binding domain.^80,81^ Missense mutations fundamentally alter the binding and transactivating abilities of the p53 protein and may contribute to tumorigenesis. Mutation of the p53 protein has been reported in more than 50% of human cancers.^82^ The p53 protein thus shows promising potential in the treatment of oncological diseases, but targeting the *TP53* gene is quite difficult.^83^ Therefore, newly developed methods of anticancer therapy focus on alternative drugs aimed at restoring the function of mutant p53 proteins^84,85^ or repressing the activity of negative regulators of p53.^86^ Negative regulators include proteins p53-induced ubiquitin-protein ligase (PIRH2),^87^ constitutive photomorphogenesis protein 1 (COP1),^88^ ADP-ribosylation factor 1 binding protein 1 (ARF-BP1),^89^ MDMX, or the main negative regulator of p53 protein, MDM2.^90^ Inhibition of the activity of negative regulators could ensure an increase in the concentration of p53-WT in cells.^83^ Moreover, the PQS in the MDM2 promoter is suggested as a target to fight malignant liposarcoma.^91^ To develop effective drugs targeting mutant p53 proteins and to prevent exacerbating the patient’s condition, a detailed understanding of these proteins’ regulatory mechanisms and binding abilities is essential. Research has focused extensively on p53 mutants, with the most frequently mutated codons observed to be R175, G245, R248, R249, R273, and R282.^92,93^

Boettcher et al. published an analysis of the DNA binding capacity of p53-WT and its mutant main mutants p53-R282W, p53-R248Q, p53-R273H, p53-R175H, p53-Y220C, and p53-M237I in TP53-K562 and TP53-MOLM13 cell lines after treatment with daunorubicin. Their results showed a strong enrichment of p53-WT protein binding, which was enhanced in the case of daunorubicin samples, and a slight enrichment in binding of the R248Q, R273H, R175H, Y220C, and M237I mutants. The results suggest that the mutant proteins retain residual DNA binding activity.^43^ Therefore, we decided to elucidate the role of other oncologically significant therapeutic targets, namely, G-quadruplexes,^94^ in the DNA binding and transactivation properties of mutated p53 proteins. Connection of the p53 regulatory network to G4s was suggested in multiple areas. The ability of p53 proteins to interact with G4 structures has been shown, as has their influence on gene expression modulation induced by p53 family proteins, including reverse modulation of p53 protein function.^69^ Moreover, the p53 protein has been demonstrated to be capable of binding to telomeric G4 directly via the DNA binding domain *in vitro*,^36^ and the binding of the full-length p53 protein or its C-terminal region to G4 in the *myc* gene promoter in the NHEIII region has been confirmed.^95^ Due to the significant regulatory properties of G4s in basic molecular–biological processes and the presence of these secondary structures in the promoters of oncogenes or telomeres,^96^ it is important to investigate their mutual interactions with p53 protein and the effect of their presence on the modulation of gene expression. As stable G4s without resolution before replication could lead to DNA damage, chromosome breaks, and cancer development,^97^ p53 mutants are also suggested to contribute to genetic DNA instability via G4 or i-motifs.^98^

Interestingly, a huge amount of the p53-ChiP and PQS overlaps was not only in the promoter regions but also in other sites of the genome. We assume that this could be due to the important role of G-quadruplexes in DNA-damage,^99,100^ where the p53 protein is one of the key regulatory proteins.^78,101^ Here, we focused on G4s in promoter regions because they represent critical regulatory elements, where p53 binding is most commonly associated with transcriptional activation or repression. By targeting these regions, we aimed to identify genes in which G4 structures may be functionally relevant in the context of p53-mediated regulation. In addition to the *in silico* approach, there are now available also experimental G4 maps based in G4-seq and/or chromatin immunoprecipitation results.^102−104^ Our analyses show that G4 structures play a significant role in the binding of mutant proteins of the p53 family that is dependent on the specific type of protein mutation in the DNA binding domain. Of particular interest were the results of the p53-R282W mutant target sites, which showed high PQS frequency in binding sites comparable to that of p53-WT binding sites. The frequency of PQS occurrence in the binding sites of p53-WT and p53-R282W proteins many times exceeded the values for other mutated proteins, including for the knockout state (Figure 1). The importance of PQS frequency in the binding sites of p53-WT and p53-R282W was confirmed, too, by comparing the frequency of PQS occurrence in reference and randomized sequences. The reference sequences showed a statistically higher frequency of PQS occurrences (at level *p* < 0.0001) in binding locations of these p53 variants compared to that in randomized sequences in both types of environments (Figure 2). The same significance was not reached for any other p53 mutant compared. Consistent with the DNA-binding ability of individual p53 variants, the most PQSs were found in the coding sequences (Figure 3). The majority representation of *in silico*-predicted G4s in protein-coding DNA regions highlights the functional importance of these structures, especially when bound by the transcriptionally active protein p53. Moreover, effect of G4s’ presence on the modulation of gene expression has been confirmed several times previously.^32,72,73,105^ A large fraction of all PQSs in p53 binding sites near the transcription start were found to be independent of DNA damage induction (presence of daunorubicin). In the case of the standard p53 protein, however, almost half of the PQS promoter was bound only under stress conditions, when the cells were treated with daunorubicin (Figure 4), which again indicates the preservation of the function of the transcription factor proteins p53-WT and p53-R282W (and possibly also of p53-Y220C).

In addition to the line K562, we have analyzed the cell line MOLM13 (Figure S3) to see if there would be a significant difference between the binding sites in the two lines. As in the case of the K562 cell line, the p53-WT and p53-R282W proteins exhibited high PQS frequency at p53 binding sites, along with the p53-Y220C protein, which in K562 exhibited high PQS frequency in a DNA damage-inducing environment. Next, promoter regions in the MOLM13 line were analyzed (Figure S4), and genes binding the p53 protein were distinguished in the K562 line only, in the MOLM13 line only, and in both lines simultaneously (Table S5). Although many genes are common to both lineages, genes bound specifically in only one lineage were also found. However, it is important to emphasize that the interaction of some important protein-coding genes with p53 proteins has been demonstrated in both lines (e.g., *MDM2, BBC3, ASTN2, GDF15, PCNA, INPP5B*, and *TRIM32*). Most of the sites found correspond to well-known and long-studied p53 protein responsive sites, so we can assume that key p53 protein binding sites are often associated with sequences capable of forming G-quadruplexes. Moreover, very well-known and characterized MDM2 and BBC3 promoter regions were found also in G4-Seq^106^ or BG4-chip-seq data sets,^45^ pointing to the cross talk between G4s and p53 target sites. Notably, some of these PQSs were also bound by the p53-R282W mutant protein regardless of the presence or absence of the inducing agent. This suggests that the p53-R282W mutant protein can bind to target sequences of the standard p53 protein near the transcription start of some genes, stabilize G4, and thus modulate the expression of these genes independently of the induction of DNA damage. PQSs in the promoter regions were further assigned to genes whose expression could be affected by the presence of G4 (Figure S2). This resulted in an extensive list of p53 REs near G4 structures (for p53-WT and p53-R282W), thus combining two important targets of newly developed therapeutic approaches in cancer treatment and underscoring the influence of their mutual interactions on the modulation of gene expression (Table S4).

Considering the results of the *in silico* analyses, we investigated the influence *in vivo* of the presence of PQSs (or the confirmed G4 structures near the RE) on the transactivation potential of p53-R282W compared to the p53-WT protein. For this purpose, we used the yeast isogenic system, which had been used previously to study the modulation of gene expression induced by proteins of the p53 family,^107,108^ including within the p53 RE near G4-prone DNA sequences.^72,109^ Although the presence of G4 located both upstream and downstream of p53 RE has been analyzed in this way several times, this has never been done for full sequences derived directly from the promoter regions of protein-coding genes. To prepare the yeast constructs, sequences were taken from promoter regions of the *BBC3* (BBC3-01 PQS) and *MDM2* (MDM2 PQS) genes where G4 presence was confirmed via the ThT assay and CD spectroscopy (Figures 5 and6). The presence of G4 can increase basal gene expression,^71^ but it also can cause transcriptional repression in the presence of a transcription factor.^72,73,109^ The results obtained in the yeast expression system point to a complex mechanism of gene expression regulation of p53-WT and p53-R282W proteins, which depends not only on the specific mutation of the protein but also on the protein concentration in cells and their localization. This regulation becomes important especially at lower protein concentrations, as was observed at time 06:00 (Figure 7A,E). Meanwhile, the results show an unmistakable influence of G4 in this regulation (Figure 7).

The differences in reporter gene expression in MDM2_RE and MDM2 versus BBC3_RE and BBC3 strains were more pronounced in the p53-R282W mutant, although its overall level of transcriptional activation was lower than that of p53-WT. The results suggest that the presence of p53-WT protein or its mutant p53-R282W can promote the expression of the negative regulator MDM2 that sets off a well-known feedback loop.^110^ The presence of G4 located downstream of the RE of the p53 protein (BBC3 strain) resulted in the repression of gene expression compared to the control strain BBC3_RE without the G4 structure for both p53 proteins. Results are consistent with previously published studies,^72,73,109^ where the Kaposi’s sarcoma-associated herpesvirus-derived G4 sequence was located directly downstream the RE without a spacer or sequence overlap and showed a high potential for G4 formation. Our data further support the assumption that the p53-R282W protein exhibits a partial loss of p53-WT function. Interestingly, G4 presented upstream from the RE of p53 protein (the MDM2 strain) supported its transactivation potential against the MDM2_RE control strain at lower concentrations of p53-R282W expression. Although the p53-WT protein did not show such a significant increase in transactivation at lower protein concentrations, both proteins showed similar profiles of transcriptional modulation at high concentrations. The results again point to a complex process of transcription regulation, as previous works pointed to the repression of transcription induced by the p53 protein even under the assumption of a G4 structure located upstream of the RE, although that did not reach such significant statistical significance as when G4 was located downstream.^72,73^ In the MDM2 and MDM2_RE strains, high concentrations of p53 proteins could lead to full saturation of the RE by proteins and possible binding of both proteins to the G4 structure, leading to its subsequent stabilization and repression of transcription.

Contrary to previously published studies, our results point to complex regulatory pathways based on the RE in association with the G4-forming sequences from real promoter regions of the human genome and open the possibility to manipulate these for effective p53 protein transcription regulation.

## Data Availability

All data available in the manuscript and supplementary files.

## Supporting Information Available

Script S1: Python code for ChIP-seq and PQS analyses overlap analyses “Intersection.py”. Script S2: Python code for overlaps with annotations “Intersection annotation.py”. Script S3: Python code for with promoters “Intersection promoter.py”. Table S1: Statistical information on input data from ChIPped p53 seq for DMSO-treated cells. Table S2: Statistical information on input data from ChIPped p53 seq for daunorubicin-treated cells. Table S3: Results from analysis of p53 protein binding sites by the G4Hunter software. Table S4: Sequences of RE and PQS binding sites of p53-WT and p53-R282W in those genes found. Table S5: Overview of genes with p53 binding sites associated with PQS in K562 and MOLM13 cell lines. Table S6: The p53 ChIP-ed promoters overlay with experimental data sets G4-seq analyses and BG4-chip-seq in K562 cells Figure S1: Graphical summary of the count of binding sites of individual p53 mutants and p53-WT. Figure S2: Representation of PQS in p53 protein binding sites in the promoter regions of individual genes. Figure S3: Frequency of PQS in binding sites of standard and mutant p53 protein in the MOLM13 cell line. Figure S4: Representation of PQS in p53 protein binding sites in the promoter regions of individual genes in cell line MOLM13 (PDF)
