# Supplementary material for: Chromatin Immunoprecipitation Reveals p53 Binding to G-Quadruplex DNA Sequences in Myeloid Leukemia Cell Lines
Source: ACS Bio Med Chem Au. 2025 Feb 12;5(2):283–98. doi: 10.1021/acsbiomedchemau.4c00124 (PMC12006861; doi:10.1021/acsbiomedchemau.4c00124)
Supplement: Supplementary file 1 — bg4c00124_si_001.pdf [file bg4c00124_si_001.pdf]

Chromatin immunoprecipitation reveals p53 binding to G-quadruplex DNA sequences in myeloid leukemia cell lines

Libuše Kratochvilová[1, 2], Alessandra Dinová[1], Natália Valková[1], Michaela Dobrovolná[1,2], Pedro A. Sánchez-Murcia,[3,4] and Václav Brázda[1,2*]

[1] Institute of Biophysics of the Czech Academy of Sciences, Královopolská 135, 612 65 Brno, Czech Republic; kratochvilova@ibp.cz (L.K.); dinovaalessandra@gmail.com (A.D.), nataliabohalova@gmail.com (N.D.), dobrovolna@ibp.cz (M.D.); vaclav@ibp.cz (V.B.)
[2] Department of Food Chemistry and Biotechnology, Faculty of Chemistry, Brno University of Technology, Purkyňova 118, 612 00 Brno, Czech Republic
[3] Laboratory of Computer-Aided Molecular Design, Division of Medicinal Chemistry, Otto-Loewi Research Center, Neue Stiftingtalstr. 6/III A-8010 Graz, Austria
[4] BioTechMed-Graz, Mozartgasse 12/II A-8010 Graz, Austria

* To whom correspondence should be addressed. Tel: +420 541 517 231; Fax: +420 541 211 293; Email: vaclav@ibp.cz

Supplementary data

Script S1: Intersection.py

```
import csv
import os
from typing import List
import sys
import pandas as pd

class G4Hunter():
    def __init__(self, position: int, len: int, score: float, sequence: str, sub_score: 
str) -> None:
        self.g4_start: int = position
        self.g4_end: int = position + len
        self.g4_len: int = len
        self.sequence: str = sequence
        self.score: float = score
        self.sub_score: str = sub_score

class P53():
    def __init__(self, start: int, end: int, len: str) -> None:
        self.p53_start = start
        self.p53_end = end
        self.p53_len = len

class Intersection():
    def map_file_names(self, g4_dir_path: str, p53_dir_path: str) -> List[List[str]]:
        """
        MAPUJE SÚBORY V ZLOŽKE S VÝSLEDKAMI Z G4HUNTERA NA SÚBORY V ZLOŽKE P53 RE 
(NÁZVY SÚBOROV SA MUSIA ZHODOVAŤ)
        """
        g4_file_names = next(os.walk(g4_dir_path))[2]
        p53_file_names = next(os.walk(p53_dir_path))[2]

        pairs = []

        for g4_name in g4_file_names:
            for p53_name in p53_file_names:
                if g4_name == p53_name:
                    pairs.append([f'{g4_dir_path}/{g4_name}', 
f'{p53_dir_path}/{p53_name}'])

        return pairs


    def load_g4hunter_results(self, folder_path: str) -> List[G4Hunter]:
        """
        NAČÍTA DÁTA Z CSV GROUPED SÚBORU S VÝSLEDKAMI 64HUNTER ANALÝZY A TRANSFORMUJE 
ICH DO G4HUNTER CLASS
        """
        loaded_results: List[G4Hunter] = []

        with open(folder_path, 'r') as file:
            reader = csv.reader(file, delimiter='\t')
            next(reader)

            for result in reader:
                loaded_results.append(
                    G4Hunter(
                        position=int(result[1]), len=int(result[2]), 
score=float(result[3]), sequence=result[5], sub_score=result[6]
                    )
                )
        return loaded_results

    def load_p53_results(self, folder_path: str) -> List[P53]:
        """
         NAČÍTA DÁTA Z CSV SÚBORU S VÝSLEDKAMI ChIP-Seq ANALÝZY A TRANSFORMUJE ICH DO 
P53 CLASS
```

```
        """
        loaded_results: List[P53] = []

        with open(folder_path, 'r') as file:
            reader = csv.reader(file, delimiter=',')
            next(reader)

            for result in reader:
                loaded_results.append(
                    P53(
                        start=int(result[1]), end=int(result[2]), len=result[3],
                    )
                )
        return loaded_results

    def intersect(self, g4s: List[G4Hunter], p53s: List[P53], file_name: str) -> None:
        """
        FUNKCIA HĽADÁ SAMOTNÝ PREKRYV SEKVENCIÍ P53 RE A G4
        """
        intersections: List[dict] = []

        for g4 in g4s:
            for p53 in p53s:
                if (p53.p53_start < g4.g4_start and g4.g4_start < p53.p53_end) or
(p53.p53_start >= g4.g4_start and p53.p53_end <= g4.g4_end) or (p53.p53_start <
g4.g4_end and p53.p53_end > g4.g4_end):
                    intersections.append({**g4.__dict__, **p53.__dict__, 'file_name':
file_name})

        return intersections

    def do_magic(self, g4_folder: str, p53_folder: str, output:str):
        """
        FUNKCIA POSTUPNE VOLÁ VYŠŠIE ZADEFINOVANÉ FUNKCIE, VÝSLEDOK UKLADÁ DO CSV
SÚBORU
        """
        pairs = self.map_file_names(g4_folder, p53_folder)

        result_collector = []

        for index, pair in enumerate(pairs):
            file_name = pair[0].split("/")[-1]

            print(f'ANALYSING FILE INDEX {index + 1}/{len(pairs)} -> {file_name}')

            g4s = self.load_g4hunter_results(pair[0])
            p53s = self.load_p53_results(pair[1])

            result_collector.extend(self.intersect(g4s, p53s, file_name))

        pd.DataFrame(data=result_collector).to_csv(output)


def main(argv):
    Intersection().do_magic(argv[0],argv[1],argv[2])
if __name__ == "__main__":
    main(sys.argv[1:])
```

Script S2: Intersection annotation.py

```
import csv
import os
from typing import List
import pandas as pd
import sys

class G4Hunter():
    def __init__(self, g4_start: int, g4_end: int, g4_len: int, score: float, sequence: 
str, sub_score: str, p53_start: int, p53_end:int, p53_len:int) -> None:
        self.g4_start: int = g4_start + 1
        self.g4_end: int = g4_end
        self.g4_len: int = g4_len
        self.sequence: str = sequence
        self.score: float = score
        self.sub_score: str = sub_score
        self.p53_start: int = p53_start + 1
        self.p53_end: int = p53_end
        self.p53_len: int = p53_len

class annotation():
    def __init__(self, start: int, end: int, strand: str, gene_name: str, gene_type: 
str, source: str) -> None:
        self.feature_start: int = start
        self.feature_end: int = end
        self.feature_strand: str = strand
        self.gene_name: str = gene_name
        self.gene_type: str = gene_type
        self.source: str = source

class Intersection():
    def map_file_names(self, g4_dir_path: str, annotation_dir_path: str) -> 
List[List[str]]:
        """
        MAPUJE SÚBORY V ZLOŽKE S VÝSLEDKAMI PREDCHÁDZAJÚCEJ ANALÝZY NA SÚBORY V ZLOŽKE 
S GENCODE ANOTÁCIAMI ĽUDSKÉHO GENÓMU (NÁZVY SÚBOROV SA MUSIA ZHODOVAŤ)
        """
        g4_file_names = next(os.walk(g4_dir_path))[2]
        annotation_file_names = next(os.walk(annotation_dir_path))[2]

        pairs = []

        for g4_name in g4_file_names:
            for annotation_name in annotation_file_names:
                if g4_name == annotation_name:
                   pairs.append([f'{g4_dir_path}/{g4_name}', 
f'{annotation_dir_path}/{annotation_name}'])

        return pairs


    def load_g4hunter_results(self, folder_path: str) -> List[G4Hunter]:
        """
        NAČÍTA DÁTA Z CSV SÚBORU S VÝSLEDKAMI PRECHÁDZAJÚCEJ ANALÝZY A TRANSFORMUJE ICH 
DO G4HUNTER CLASS
        """

        loaded_results: List[G4Hunter] = []

        with open(folder_path, 'r') as file:
            reader = csv.reader(file, delimiter=',')
            next(reader)

            for result in reader:
                loaded_results.append(
                    G4Hunter(
```

```
                        g4_start=int(result[1]), g4_end=int(result[2]),
g4_len=int(result[3]), sequence=result[4], score=float(result[5]), sub_score=result[6],
p53_start=int(result[7]), p53_end=int(result[8]), p53_len=int(result[9])
                    )
                )
        return loaded_results

    def load_annotation_results(self, folder_path: str) -> List[annotation]:
        """
        NAČÍTA DÁTA Z CSV SÚBORU S GENCODE ANOTÁCIAMI ĽUDSKÉHO GENÓMU A TRANSFORMUJE
ICH DO ANNOTATION CLASS
        """
        loaded_results: List[annotation] = []

        with open(folder_path, 'r') as file:
            reader = csv.reader(file, delimiter=',')
            next(reader)

            for result in reader:
                loaded_results.append(
                    annotation(
                        start=int(result[1]), end=int(result[2]), strand=result[4],
gene_type=result[10], gene_name=result[11], source=result[5]
                    )
                )
        return loaded_results

    def intersect(self, g4s: List[G4Hunter], annotations: List[annotation], file_name:
str) -> None:
        """
        FUNKCIA HĽADÁ SAMOTNÝ PREKRYV POZÍCIÍ P53 RE A ANOTÁCIÍ
        """

        intersections: List[dict] = []

        for g4 in g4s:
            for annotation in annotations:
                if ((g4.score < 0 and annotation.feature_strand == "-") or (g4.score >
0 and annotation.feature_strand == "+")) and ((annotation.feature_start < g4.p53_start
and g4.p53_start < annotation.feature_end) or (annotation.feature_start > g4.p53_start
and annotation.feature_start < g4.p53_end) or (annotation.feature_start >= g4.p53_start
and annotation.feature_end <= g4.p53_end) or (annotation.feature_start < g4.p53_end and
annotation.feature_end > g4.p53_end)):
                    intersections.append({**g4.__dict__, **annotation.__dict__,
'file_name': file_name})

        return intersections

    def do_magic(self, g4_folder: str, annotation_folder: str, output:str):
        """
        FUNKCIA POSTUPNE VOLÁ VYŠŠIE ZADEFINOVANÉ FUNKCIE, VÝSLEDOK UKLADÁ DO CSV
SÚBORU
        """
        pairs = self.map_file_names(g4_folder, annotation_folder)

        result_collector = []

        for index, pair in enumerate(pairs):

            file_name = pair[0].split("/")[-1]

            print(f'ANALYSING FILE INDEX {index + 1}/{len(pairs)} -> {file_name}')

            g4s = self.load_g4hunter_results(pair[0])
            annotations = self.load_annotation_results(pair[1])

            result_collector.extend(self.intersect(g4s, annotations, file_name))
```

```
        pd.DataFrame(data=result_collector).to_csv(output)

def main(argv):
    Intersection().do_magic(argv[0],argv[1],argv[2])
if __name__ == "__main__":
    main(sys.argv[1:])
```

Script S3: Intersection promoter.py

```
import csv
import os
from typing import List
import pandas as pd
import sys

class G4Hunter():
    def __init__(self, g4_start: int, g4_end: int, g4_len: int, score: float, sequence: str, sub_score: str, p53_start: int, p53_end:int, p53_len:int) -> None:
        self.g4_start: int = g4_start + 1
        self.g4_end: int = g4_end
        self.g4_len: int = g4_len
        self.sequence: str = sequence
        self.score: float = score
        self.sub_score: str = sub_score
        self.p53_start: int = p53_start + 1
        self.p53_end: int = p53_end
        self.p53_len: int = p53_len

class annotation_F():
    def __init__(self, start: int, strand: str, gene_name: str, gene_type: str, source: str) -> None:
        self.feature_start: int = start - 1000
        self.feature_end: int = start + 100
        self.feature_strand: str = strand
        self.gene_name: str = gene_name
        self.gene_type: str = gene_type
        self.source: str = source

class annotation_R():
    def __init__(self, end: int, strand: str, gene_name: str, gene_type: str, source: str) -> None:
        self.feature_start: int = end - 100
        self.feature_end: int = end + 1000
        self.feature_strand: str = strand
        self.gene_name: str = gene_name
        self.gene_type: str = gene_type
        self.source: str = source

class Intersection():
    def map_file_names(self, g4_dir_path: str, annotation_dir_path: str) -> List[List[str]]:
        """
        MAPUJE SÚBORY V ZLOŽKE S VÝSLEDKAMI PREDCHÁDZAJÚCEJ ANALÝZY NA SÚBORY V ZLOŽKE S GENCODE ANOTÁCIAMI ĽUDSKÉHO GENÓMU (NÁZVY SÚBOROV SA MUSIA ZHODOVAŤ)
        """

        g4_file_names = next(os.walk(g4_dir_path))[2]
        annotation_file_names = next(os.walk(annotation_dir_path))[2]

        pairs = []

        for g4_name in g4_file_names:
            for annotation_name in annotation_file_names:
                if g4_name == annotation_name:
                    pairs.append([f'{g4_dir_path}/{g4_name}', f'{annotation_dir_path}/{annotation_name}'])

        return pairs


    def load_g4hunter_results(self, folder_path: str) -> List[G4Hunter]:
        """
        NAČÍTA DÁTA Z CSV SÚBORU S VÝSLEDKAMI PRECHÁDZAJÚCEJ ANALÝZY A TRANSFORMUJE ICH DO G4HUNTER CLASS
        """
```

```
        loaded_results: List[G4Hunter] = []

        with open(folder_path, 'r') as file:
            reader = csv.reader(file, delimiter=',')
            next(reader)

            for result in reader:
                loaded_results.append(
                    G4Hunter(
                        g4_start=int(result[1]), g4_end=int(result[2]),
g4_len=int(result[3]), sequence=result[4], score=float(result[5]), sub_score=result[6],
p53_start=int(result[7]), p53_end=int(result[8]), p53_len=int(result[9])
                    )
                )
        return loaded_results

    def load_annotation_results(self, folder_path: str):
        """
        NAČÍTA DÁTA Z CSV SÚBORU S GENCODE ANOTÁCIAMI ĽUDSKÉHO GENÓMU A TRANSFORMUJE
ICH DO ANNOTATION_R CLASS PRE REVERSE REŤAZEC DNA A ANNOTATION_F CLASS PRE FORWARD
REŤAZEC DNA
        """
        loaded_results_R: List[annotation_R] = []
        loaded_results_F: List[annotation_F] = []

        with open(folder_path, 'r') as file:
            reader = csv.reader(file, delimiter=',')
            next(reader)

            for result in reader:
                if result[6] == "transcript":
                    loaded_results_F.append(
                        annotation_F(
                            start=int(result[1]), strand=result[4],
gene_type=result[10], gene_name=result[11], source=result[5]
                        )
                    )

                    loaded_results_R.append(
                    annotation_R(
                        end=int(result[2]), strand=result[4], gene_type=result[10],
gene_name=result[11], source=result[5]
                    )
                )
        return loaded_results_F,loaded_results_R

    def intersect(self, g4s: List[G4Hunter], annotations_F: List[annotation_F],
annotations_R: List[annotation_R], file_name: str) -> None:
        """
        FUNKCIA HĽADÁ SAMOTNÝ PREKRYV POZÍCIÍ P53 RE A PROMÓTOROV ANOTOVANÝCH GÉNOV NA
ZÁKLADE ORIENTÁCIE REŤAZCA
        """
        intersections: List[dict] = []

        for g4 in g4s:
            for annotation_F in annotations_F:
                if (g4.score > 0 and annotation_F.feature_strand == "+") and
((annotation_F.feature_start < g4.p53_start and g4.p53_start <
annotation_F.feature_end) or (annotation_F.feature_start > g4.p53_start and
annotation_F.feature_start < g4.p53_end) or (annotation_F.feature_start >= g4.p53_start
and annotation_F.feature_end <= g4.p53_end) or (annotation_F.feature_start < g4.p53_end
and annotation_F.feature_end > g4.p53_end)):
                    intersections.append({**g4.__dict__, **annotation_F.__dict__,
'file_name': file_name})
            for annotation_R in annotations_R:
```

```
                if (g4.score < 0 and annotation_R.feature_strand == "-") and
((annotation_R.feature_start < g4.p53_start and g4.p53_start <
annotation_R.feature_end) or (annotation_R.feature_start > g4.p53_start and
annotation_R.feature_start < g4.p53_end) or (annotation_R.feature_start >= g4.p53_start
and annotation_R.feature_end <= g4.p53_end) or (annotation_R.feature_start < g4.p53_end
and annotation_R.feature_end > g4.p53_end)):
                    intersections.append({**g4.__dict__, **annotation_R.__dict__,
'file_name': file_name})

        return intersections

    def do_magic(self, g4_folder: str, annotation_folder: str, output:str):
        """
        FUNKCIA POSTUPNE VOLÁ VYŠŠIE ZADEFINOVANÉ FUNKCIE, VÝSLEDOK UKLADÁ DO CSV
SÚBORU
        """
        pairs = self.map_file_names(g4_folder, annotation_folder)

        result_collector = []

        for index, pair in enumerate(pairs):
            file_name = pair[0].split("/")[-1]

            print(f'ANALYSING FILE INDEX {index + 1}/{len(pairs)} -> {file_name}')

            g4s = self.load_g4hunter_results(pair[0])
            annotations = self.load_annotation_results(pair[1])

            result_collector.extend(self.intersect(g4s, annotations[0], annotations[1],
file_name))

        pd.DataFrame(data=result_collector).to_csv(output)

def main(argv):
    Intersection().do_magic(argv[0],argv[1],argv[2])
if __name__ == "__main__":
    main(sys.argv[1:])
```

Table S1: Statistical information on input data from ChIPped p53-seq for DMSO-treated K562 cells.

Chromosome	Knock-out			p53-WT			p53-R282W			p53-R175H			p53-220			p53-237			p53-R248			p53-273			p53-WT unique			p53-R282W unique			Intersect		
	Count	Length	GC %	Count	Length	GC %	Count	Length	GC %	Count	Length	GC %	Count	Length	GC %	Count	Length	GC %	Count	Length	GC %	Count	Length	GC %	Count	Length	GC %	Count	Length	GC %	Count	Length	GC %
**1**	5	2 469	37	33	11 913	41	32	10 220	41	23	9 439	37	24	9890	38	15	6742	37	22	9230	37	23	9829	37	11	2 964	48	9	2 310	48	18	4 837	40
**2**				4	1 174	47	3	611	44																3	918	48	2	411	43	1	200	47
**3**	1	582	23	7	2 109	42	6	1 748	41	2	820	37	3	1 111	41	2	887	40	3	1 163	42	2	819	36	4	1 046	48	3	682	47	2	536	50
**4**	1	498	48	12	3 592	45	10	2 736	43	8	2 667	41	9	3 097	40	3	1 226	37	5	2 008	35	6	2 267	39	5	1 326	49	3	619	43	6	1 465	41
**5**	3	1 414	40	10	4 560	45	15	4 925	44	5	3 005	42	7	4 155	43	4	2 685	41	3	2 726	41	4	3 044	42	3	819	50	7	1 664	45	5	1 259	47
**6**				12	3 344	48	8	2 244	50				1	266	39										6	1 463	49	2	434	53	6	1 538	48
**7**				7	1 967	50	3	809	55																6	1 726	50	2	487	57	1	241	54
**8**				4	1 145	47	5	1 244	47				1	280	46										2	495	48	3	723	47	2	508	47
**9**				4	1 297	54	6	1 465	57																1	289	55	3	758	57	3	707	56
**10**				8	2 349	48	6	1 744	47	3	1 098	46	4	1 444	45	3	1 175	44	3	1 171	45	3	1 255	45	3	799	53	1	219	57	5	1 370	46
**11**				4	1 261	52	8	1 799	53	4	3 570	51	1	789	46	1	409	47	1	385	47	1	473	46	2	721	50	6	1 314	51	2	457	56
**12**				2	774	51	8	2 117	56	1	474	59	2	684	56	1	373	60	1	414	60	1	457	60				6	1 485	57	2	617	52
**13**				5	1 583	52	2	505	49				2	520	51										4	1 177	55	1	238	48	1	267	49
**14**				2	532	47	4	901	58																1	287	48	3	709	62	1	192	45
**15**				2	710	67	5	1 448	56																			3	738	48	2	625	66
**16**	1	333	36	19	7 871	38	22	6 512	39	13	6 119	37	17	8 512	37	13	5 924	36	13	6 729	36	16	7 553	36	5	1 242	39	5	1 140	43	15	4 571	38
**17**				7	2 172	44	11	3 195	45	6	1 849	40	7	2 348	40	5	1 803	40	5	1 827	40	6	2 107	41	1	269	51	5	1 379	45	6	1 738	44
**18**	1	459	49	3	1 039	50	3	1 122	49	2	774	49	2	889	49	1	549	48	1	589	48	1	576	48							2	474	51
**19**				5	1 738	63	7	2 434	62				2	621	69							1	282	70	1	288	48	3	788	56	4	1 323	66
**20**				1	232	55	4	939	56	1	249	41	1	257	40													3	685	56	1	232	55
**21**				8	2 678	58	5	1 777	65	4	1 661	67	4	2 116	64	2	624	63	4	1 936	65	5	2 034	66	4	975	47	1	275	51	4	1 429	66
**22**				1	291	54	3	722	57	1	262	53	1	303	55				1	310	56	1	233	53				2	475	59	1	247	55
**X**				9	2 612	50	3	782	48	1	298	51	1	349	50				1	308	50	1	255	50	6	1 743	51				3	761	47
**Y**				3	850	45	3	753	45	3	830	45	3	943	45	2	490	43	3	888	45	3	910	45							3	753	45

Table S2: Statistical information on input data from ChIPped p53 seq for daunorubicin-treated K562 cells.

Chromosome	Knock-out			p53-WT			p53-R282W			p53-R175H			p53-220			p53-237			p53-R248			p53-273			p53-WT unique			p53-R282W unique			Intersect		
	Count	Length	GC %	Count	Length	GC %	Count	Length	GC %	Count	Length	GC %	Count	Length	GC %	Count	Length	GC %	Count	Length	GC %	Count	Length	GC %	Count	Length	GC %	Count	Length	GC %	Count	Length	GC %
**1**	15	7792	37	77	23 307	45	74	20 712	44	32	11 157	38	39	12 002	40	25	8 871	37	22	9 611	37	24	9 315	37	33	8 599	50	31	7 435	47	30	6 881	46
**2**				29	7 638	47	26	6 075	47				4	1 099	47										20	5 298	48	17	3 867	48	9	2 008	45
**3**	1	542	22	24	6 469	46	19	4 988	45	3	1 113	45	5	1 696	44	2	913	43	2	890	38	2	905	42	13	2 961	48	8	1 778	47	10	2 531	48
**4**	2	945	41	31	8 206	46	19	5 121	47	8	2 620	46	9	2 785	41	7	2 127	35	5	1 877	46	7	2 374	39	19	4 585	47	7	1 760	50	10	2 237	44
**5**	4	2334	41	31	10 648	45	32	9 402	45	5	3 459	41	10	4 431	43	4	3 039	41	4	2 789	41	4	3 091	41	15	3 861	48	15	3826	47	13	3 261	46
**6**				38	10 655	48	28	7 437	47				9	2 133	47	2	495	41							20	5 079	49	10	2 080	45	18	4 842	47
**7**				33	8 580	48	21	5 180	52				4	993	53										23	5 704	48	11	2 487	56	10	2 490	48
**8**				35	9 509	48	24	5 969	47				4	982	55										20	5 078	48	9	2 200	45	15	3 615	48
**9**				29	7 788	51	23	5 729	53				5	1 256	53										17	4 247	50	11	2 494	52	12	3 148	54
**10**	1	629	49	24	6 593	48	16	4 079	49	6	1 849	47	6	1 950	45	4	1 468	44	3	1 265	44	4	1 334	44	13	3 180	48	5	1 137	53	10	2 322	47
**11**				23	6 489	48	19	4 891	50	1	504	47	3	782	48	3	903	58	1	454	47	1	380	47	15	4 088	47	11	2 870	50	8	1 965	50
**12**	1	292	61	25	6 552	49	25	6 719	51	2	668	57	2	722	52	1	451	60	1	429	60	1	476	60	12	2 878	49	12	3 303	51	12	2 700	50
**13**				13	3 876	52	9	2 277	51				5	1 397	54										6	1 509	51	2	445	44	7	1 726	53
**14**				18	4 589	49	15	3 720	49				1	227	50										8	2 088	47	5	1 275	46	10	2 275	51
**15**				17	4 247	49	13	3 429	50				2	502	66										11	2 540	44	7	1 786	46	6	1 536	55
**16**	5	2363	37	32	11 470	39	36	10 063	41	17	7 524	36	22	8 845	37	15	7 446	36	15	7 437	36	14	7 439	37	9	2 091	45	12	2 640	48	19	4 916	40
**17**	2	760	40	22	6 489	45	24	6 565	48	10	3 026	41	9	2 757	41	5	1 790	40	4	1 842	41	8	2 450	41	5	1 207	43	7	1 711	55	15	3 639	46
**18**	1	543	48	10	2 928	50	8	2 159	49	1	648	48	1	651	49	1	677	49	1	624	49	2	827	55	5	1 156	50	3	608	48	4	919	50
**19**				17	4 527	58	21	5 728	57	1	271	69	3	926	69				1	287	69	1	363	70	9	2 053	56	13	3 144	55	8	2 173	60
**20**	1	279	40	8	1 940	49	9	2 358	53	3	693	41	2	429	48	2	440	41	1	271	40	2	415	41	4	888	45	6	1 406	51	3	826	56
**21**	3	1534	67	16	5 300	54	18	4 814	53	4	1 871	64	5	1 966	62	5	1 339	62	4	1 893	64	4	1 729	63	5	1 371	46	7	1 700	50	8	1 894	47
**22**				6	1 641	53	8	2 055	55				2	473	55										3	718	56	5	1 232	58	3	823	50
**X**				19	5 337	50	7	1 835	48	1	228	50	4	909	48										13	3 366	52	1	202	52	6	1 627	47
**Y**	1	342	43	4	979	45	3	734	45	5	1 318	46	3	828	45	2	600	44	4	1 175	45	3	870	45	1	231	47				2	496	45

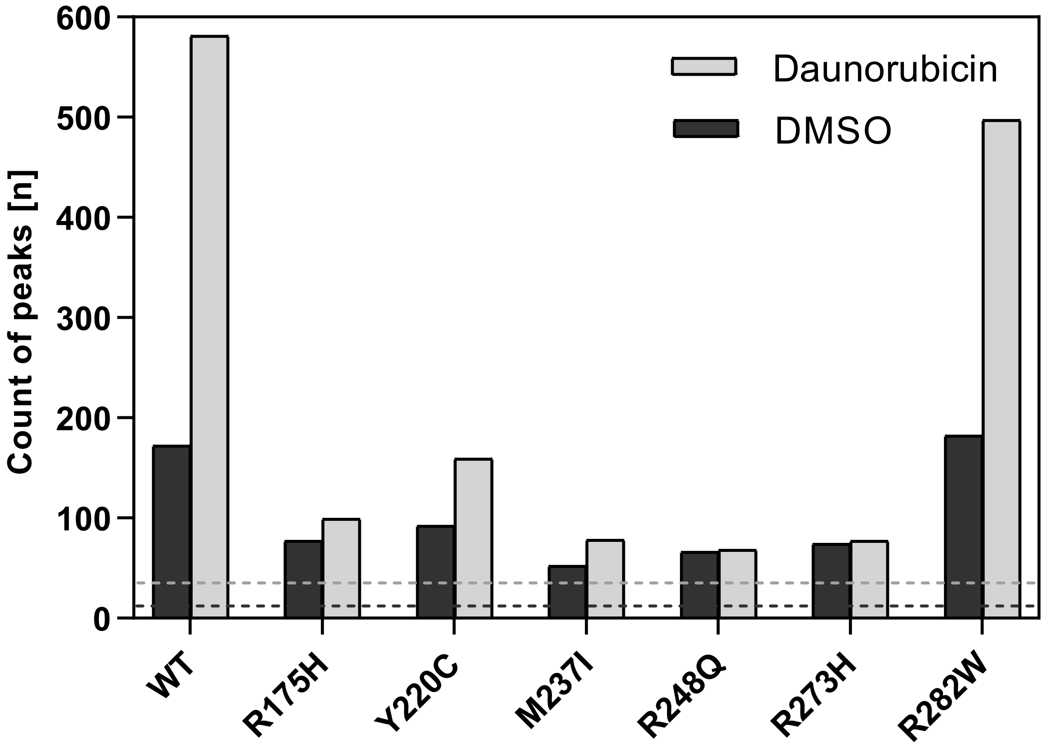

Figure S1: Graphical summary of the count of binding sites of individual p53 mutants and p53-WT. Comparison as to the number of p53 binding sites found in the environment of both chemicals. The black broken line indicates the number of peaks in the knock-out condition in DMSO-treated K562 cells. The grey broken line indicates the number of peaks in the knock-out condition in cells treated with daunorubicin.

Table S3: Results from analysis of p53 protein binding sites by G4Hunter software.

	ChIPped p53 seq			PQS count						PQS length						PQS frequency / 1 000 bp					
	Count	Length	GC %	Total	1.2-1.4	1.4-1.6	1.6-1.8	1.8-2.0	2.0+	Total	1.2-1.4	1.4-1.6	1.6-1.8	1.8-2.0	2.0+	Total	1.2-1.4	1.4-1.6	1.6-1.8	1.8-2.0	2.0+
**KO dmso**	12	5 755	38,09	0	0	0	0	0	0	0	0	0	0	0	0	0,000	0,000	0,000	0,000	0,000	0,000
**KO dauno**	37	18 355	41,30	20	10	4	3	2	1	671	277	130	108	79	77	1,090	0,545	0,218	0,163	0,109	0,054
**WT dmso**	172	57 793	46,14	194	88	46	31	14	15	10 658	2 401	1 529	1 956	570	4 202	3,357	1,523	0,796	0,536	0,242	0,260
**WT dauno**	581	165 757	47,36	567	287	131	83	33	33	18 492	7 802	4 450	3 138	1 394	1 708	3,421	1,731	0,790	0,501	0,199	0,199
**mut282 dmso**	182	52 752	47,13	228	110	47	34	16	21	12 636	2 997	1 566	2 464	667	4 942	4,322	2,085	0,891	0,645	0,303	0,398
**mut282 dauno**	497	132 039	47,83	500	253	105	63	35	44	16 674	6 834	3 522	2 467	1 515	2 336	3,787	1,916	0,795	0,477	0,265	0,333
**mut175 dmso**	77	33 115	42,34	38	13	7	5	3	10	5 964	356	234	948	139	4 287	1,148	0,393	0,211	0,151	0,091	0,302
**mut175 dauno**	99	36 949	42,18	33	14	3	7	4	5	3 261	391	98	699	253	1 820	0,893	0,379	0,081	0,189	0,108	0,135
**mut220 dmso**	92	38 574	42,23	54	24	8	8	4	10	6 847	667	258	1 436	171	4 315	1,400	0,622	0,207	0,207	0,104	0,259
**mut220 dauno**	159	50 741	44,45	112	51	27	17	8	9	3 706	1 400	885	635	327	459	2,207	1,005	0,532	0,335	0,158	0,177
**mut237 dmso**	52	22 887	39,74	7	4	1	1	1	0	223	117	33	41	32	0	0,306	0,175	0,044	0,044	0,044	0,000
**mut237 dauno**	78	30 559	40,56	25	12	4	4	2	3	844	330	125	140	79	170	0,818	0,393	0,131	0,131	0,065	0,098
**mut248 dmso**	66	29 684	40,99	27	11	4	3	3	6	2 951	305	130	108	130	2 278	0,910	0,371	0,135	0,101	0,101	0,202
**mut248 dauno**	68	30 844	41,42	21	11	4	2	2	2	702	305	130	74	79	114	0,681	0,357	0,130	0,065	0,065	0,065
**mut273dmso**	74	32 094	41,35	28	14	4	3	2	5	4 102	383	130	108	79	3 402	0,872	0,436	0,125	0,093	0,062	0,156
**mut273 dauno**	77	31 968	41,17	23	13	4	3	2	1	747	354	130	107	79	77	0,719	0,407	0,125	0,094	0,063	0,031
**WT unique dmso**	68	18 547	48,89	70	34	17	13	3	3	2 911	913	570	531	120	777	3,774	1,833	0,917	0,701	0,162	0,162
**WT unique dauno**	299	74 776	48,50	273	143	74	34	13	9	8 684	3 899	2 514	1 264	549	458	3,651	1,912	0,990	0,455	0,174	0,120
**mut282 unique dmso**	73	17 533	50,29	105	55	20	15	6	9	4 947	1 484	676	998	250	1 539	5,989	3,137	1,141	0,856	0,342	0,513
**mut282 unique dauno**	215	51 386	49,39	224	110	54	22	16	22	7 514	2 960	1 799	888	700	1 167	4,359	2,141	1,051	0,428	0,311	0,428
**Intersect dmso**	96	26 347	46,94	111	48	26	16	10	11	7 296	1 316	859	1 348	417	3 356	4,213	1,822	0,987	0,607	0,380	0,418
**Intersect dauno**	248	60 850	47,94	246	124	48	39	15	20	8 179	3 354	1 625	1 505	649	1 046	4,043	2,038	0,789	0,641	0,247	0,329

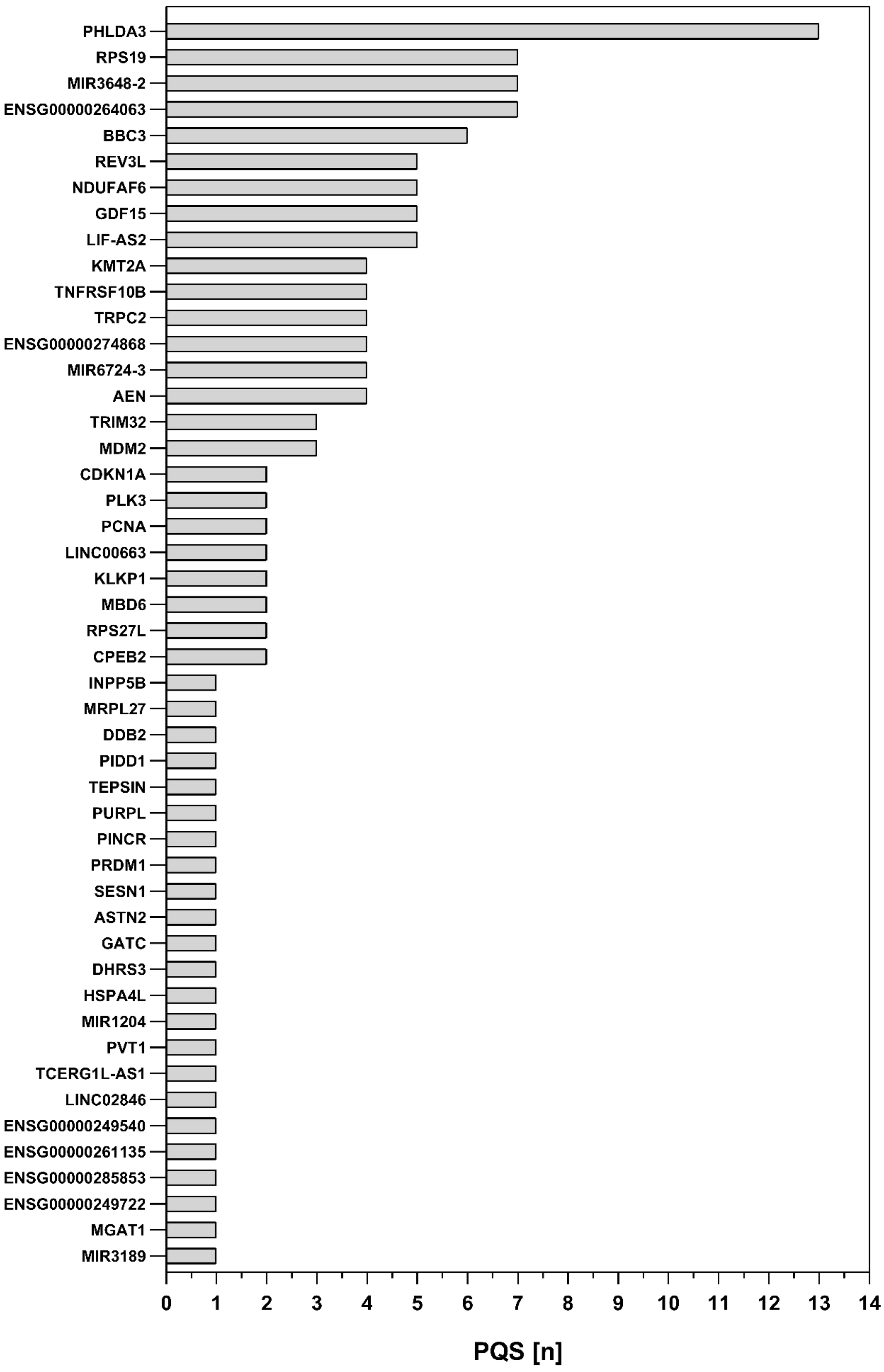

Figure S2: Representation of PQS in p53 protein binding sites in the promoter regions of individual genes.

Table S4: Sequences of RE and PQS binding sites of p53-WT and p53-R282W in those genes found.

Gene name	RE sequence	P53retriever grade	G4 sequence	G4Hunter score	Gene type	P53 protein
AEN	GGGCTTGCCCGGGCATGTGG	4	CCTGCACCGGGGCGGCGGGGTCGGGCGGGCTGAGGCCGCGTG	1,000	Protein coding	WT R282W
			TGCTCGGGGGTCCAGGGCCAGAGGGGGCGGCGC	1,303		WT
			CAGGGCCAGAGGGGGCGGCGCTGGTCG	1,111		
			CAGAGGGGGCGGCGCTGGTCGTTGGGACT	1,138		
ASTN2	CAGCATGCTGGGGCTTGTCG	4	CACCCCTGCAGGGCCGCCTTGCCCC	-1,200	Protein coding	
BBC3	CTGCAAGTCCTGACTTGTCC	3	CCCGGGCCCGCTCCAAAGCCGCCCC	-1,240	Protein coding	WT R282W
			GGCCCGCTCCAAAGCCGCCCCGCCCCGCCCCGCCCCGCCCCCGCCCGGCGGGTCCCACGCCCCGCCCCCGCGTGACGCTACGGCCCCGCCC	-1,637		
			CCCCGCCCGCCCGCCGCGGACAAGT	-1,240		
			TGCAGGCGCGCGCCGCGCCCCCCCCTACCTCCGCGCCGGG	-0,750		
			GACCCACAGATCCACACCCCCAGCGAT	-1,111		R282W
CDKN1A	GAACATGTCCCAACATGTTG	4	CACCGCACTCTGGGGAGGGGGCTGGACTGGGCACTCTTG	0,949	Protein coding	WT R282W
			TTTCAGGTGAGGAAGGGGATGGTAGGAGACAGGAGA	1,028		
	GAACATGTCCCAACATGTTG	5	TTTCAGGTGAGGAAGGGGATGGTAGGAGACAGGAGA	1,028		
			CACCGCACTCTGGGGAGGGGGCTGGACTGGGCACTCTTG	0,949		
CPEB2	AAACAAGCTT	1	TTAGAGTCTCCCTGGGGGGAAGGGGCGGGGACGCCGGAATGAATGTG	1,085	Protein coding	R282W
			GGGGACGCCGGAATGAATGTGGTGGGGG	1,407		
DDB2	GAACAAGCCCTGGGCATGTTT	3	AGGAAGGGGCGGGGTCTCCGAGACGGGTGGGGCCGG	1,556	Protein coding	WT
DHRS3	ACGCAAGCTCCCCCGGGCCTGCTC	1	TGGCTGCTGCGGCCTCCCCTCCCCCTGCGCCGTCGCGG	-0,737	Protein coding	R282W
ENSG00000249540	GGGCGTACCTAATTGAGGCATGCTT	2	CCTCACCCCAGCTGCCCTTTTGTCCCACTG	-1,233	Processed pseudogene	R282W
	CGGCTACCCACCAGGAGAAGGACTTGCCC					
	CAGCTTCCTCTCAAATGAACAGCAAGTCC	1				
ENSG00000249722	GGGCAAGCCTGGGCCTGTTT	3	TGAGTCTAAGGAAGGTGGGGGACAGGAT	1,143	lncRNA	WT
	GGATATGTCTGGGCAGGTCC	4				
	GGCAATGTCTGGGCTTGCTT	4				

GDF15	AGACCTGCCCGGACATGTCC	4	CTAGAAGCAGGGAGGGGTGGGTGAGGCTGTCACCT	0,886	Protein coding	WT R282W
			AGTGTGATGACAGCCTGGGGTGGGGGTAAGGGGGCAAAGTACAGCAC	1,128		WT
			GTATGGTGCTGATAAGGGCGTGGGGAGGCAGAGAC	1,000		
			TAAGGGCGTGGGGAGGCAGAGACAGGCAG	1,138		
	AGGGTTTCTCCATGTTGGTCAGGCTAGTCT	1	TTACTCTGCAGGCAGGGGGAGGAGGGCGGGACTGAGCAGGCGG	1,140		
	CATCTTGCCCAGACTTGTCT	3				
HSPA4L	GGACTTCCTCGCCCCGCAAAGCTTCTTT	1	AGTTGCTGGAATGCGGCGGGGTTGGGTCGGAC	1,063	Protein coding	R282W
	AAACATGTTTCCACATGTTC	4				
INPP5B	GGGCATGCTGGGACTTGCAG	4	CATTTCCCAACAGCCCCTGCGCCAC	-1,200	Protein coding	R282W
	AGCCATGTCCTAGGAGCAAAGGGACTAGGTC	1				
LIF-AS2	CGACATGTCCTGGTTTGCCC	3	AGGTCATAGGCAGTCGGGTGGGCGGGGAGGAAGGAAAGTGAAAGGTGA	1,125	lncRNA	WT
			GAAGGGCACCTGAAGATGGTGGGGG	1,240		
			GGGCACCTGAAGATGGTGGGGGCCGGGGTCAGCGGGCGAGTGAGG	1,244		
			GGGGTCAGCGGGCGAGTGAGGCTGG	1,320		
			CATGCTGGAACAGCGGGGACAGGGGCCT	0,929		
LINC02846	CAGCGTGCCCGGGCATGCCG	4	GCCCGCCGCAGCAGCCTGCAACCCCGCCCCCAGGCGCAGTGCGCGCC	-1,021	miRNA	WT
LINC00663	AGACAAGCTGTCCTGGTAGCTTGTCC	1	GCGCGCACCGCCCTGCCCCTTCCTCT	-1,192	lncRNA	R282W
			GCCCCCACCTTCAGCCTCGCTCCTCCA	-1,296		
	GAGCATGCTAGGACATGTAA	4	GCGCGCACCGCCCTGCCCCTTCCTCT	-1,192		
			GCCCCCACCTTCAGCCTCGCTCCTCCA	-1,296		
MBD6	CGGGAAGCCTTTGGGGCGGGGCAGGGCAAGTCG	1	TCTGAAAATGGGGGTAGTGTGGAGGGTGGACCAGA	1,029	Protein coding	R282W
			CCGGGAAGCCTTTGGGGCGGGGCAGGGCAAGTCGCGACTT	0,950		
	TGACATGTTGACCTGGGGAGGCGGGTCAAGTCC		TCTGAAAATGGGGGTAGTGTGGAGGGTGGACCAGA	1,029		
			CCGGGAAGCCTTTGGGGCGGGGCAGGGCAAGTCGCGACTT	0,950		
MDM2	GGTCAAGTTCAGACACGTTC	2	GGGCGGGATTGGGCCGGTTCAGTGGG	1,346	Protein coding	WT R282W
			TTCAGGGTAAAGGTCACGGGGGCCGGGGGCTGCGGGGCCGCTTCGG	1,304		R282W
			CGGGGGCTGCGGGGCCGCTTCGGCGCGGG	1,379		
	AGTCCTGACTTGTCT		TTCAGGGTAAAGGTCACGGGGGCCGGGGGCTGCGGGGCCGCTTCGG	1,304		
			CGGGGGCTGCGGGGCCGCTTCGGCGCGGG	1,379		
			GGGCGGGATTGGGCCGGTTCAGTGGG	1,346		WT R282W
MIR1204	GGGCTAGTTCCTTGCTAGGAAAAAAAAACTCT	1	TGTGTTTGTGTGTGGTGGGTGGGCGGGGGCCCTGAGGATGCAGGGA	1,109	miRNA	WT R282W
	CGACAAGTTGAGACTTGTTC	4				
MRPL27	GGGCATGTTTTAACATGTTG	5	TTTGCTCTTCACCCTACTCCCCATCCTCCTCCTCAAAG	-1,053	Protein coding	WT R282W
	TGCCCCAGCTAGTCT	2				

NDUFAF6	AAACAAGCCCGGGCA	3	AGGCCTGGGCGAGCGGAAGGGTGGGAG	1,185	Protein coding	WT
			CTGGGCGAGCGGAAGGGTGGGAGAGAGGGGCTAGGCCGGGGAGGGAAGCGCTGGGGCCGGGGCCGGCTT	1,420		
			AGGAGCCGGCGGGAGCGGGGTGCGTA	1,154		
			CGGCGGGAGCGGGGTGCGTAAGAGGCGGGCGAGGAA	1,222		
			CGGCGGGCAACGGGAAGGGCAGAGGTGGAT	1,133		
PCNA	AGGCTTTTCCTCCCGGTAGGCTTGATT	1	GTCCATGCTCCCCGCGAGGCCCGCCCCCTAGAGCAT	-1,139	Protein coding	WT
			CCCCGCCTCTTTGACTCCTGAACCC	-1,280		
	ACATATGCCCGGACTTGTTC	4	GTCCATGCTCCCCGCGAGGCCCGCCCCCTAGAGCAT	-1,139		
			CCCCGCCTCTTTGACTCCTGAACCC	-1,280		
PHLDA3	AAGCAAGCCC	1	CCCGCCCGCAGCGCATTCCTACCCT	-1,200	Protein coding	WT
			CCCCGCTTCAGCCGGCACCCGCTCC			
			CCCAGCCCGACCCGCCTCTGCCAGA	-1,240		
			CCCCTCCGCGCCGCGCACCAGCTCC			
			TCCTCCGCTCTACCCCAGCTGGCCCA	-1,154		
			CCGCTCTACCCCAGCTGGCCCAGCCC	-1,308		
			CCCACCGCCCCGCTTCAGCCGGCACCC	-1,407		
			GGCACCCGCTCCTCCGCTCTACCCCAGCT	-1,069		
			CTCTACCCCAGCTGGCCCAGCCCGACCCGCCT	-1,313		
			GCCGGCCCTCCCCTCCGCGCCGCGCACCAGCT	-1,063		
			CATTCCTACCCTGGCCGGCCCTCCCCTCCGCGCCG	-1,171		
			TGCCCCCAGCGCGCTCCGCGCCCACCGCCCCGCTTCAGCCG	-1,268		
			CTCCCTAGGCCGTGAGCCCCACCGCCCGCCCGTTCTCTTGCTCCCCTG	-1,208		
PIDD1	TGCTGGGACATGTCT	3	CCCCTCCACCGTTGCAGCCATCGCCC	-1,346	Protein coding	R282W
PINCR	TAACATGCCTGTCATAGTCC	3	GGAAGCATGGGGTGGAGCCACTGGGGATTCGTT	1,091	lncRNA	WT
	TAGCTTGTTCCTTCCAGTCC	2				
	AAACTTGTCTGAAATTATGGGAAACTTCTTA	1				
	GCCCTTGTCTGGACATGCCC	4				
PLK3	AAGGCAGCCCTCGGGCCAGGCAAGCCA	1	GTGGCCAAGGAGGGGTGAGAGGCGG	1,200	Protein coding	WT
			CAAGGAGGGGTGAGAGGCGGACAGGGATCAG	1,129		
	TAACATGCCCGGGCA	4	GTGGCCAAGGAGGGGTGAGAGGCGG	1,200		
			CAAGGAGGGGTGAGAGGCGGACAGGGATCAG	1,129		
PRDM1	GTGCAAGTCTGGACATGTTT	4	TACTTTAGGACTTGGAGGGTTGGGGGTGGAGGATGTTTGCATAGTT	0,978	Protein coding	WT R282W
PURPL	AAACAAGTTAGTTTTTTTTAAGAGATGCCA	1	GGGGCAAGTGGGTGGAGCCATGAGG	1,240	lncRNA	WT
	GGGCTTGTCTGGGCATGCCC	5				
	TGACATGCTCACTGGGGCAAGTGG	1				

PVT1	GGGCTAGTTCCTTGCTAGGAAAAAAAAACTCT	1	TGTGTTTGTGTGTGGTGGGTGGGCGGGGGCCCTGAGGATGCAGGGA	1,109	lncRNA	WT
	AGGCAATTCTGGATTTATGAACTTGATC					
	AGACTTGCCTGGGCTTGTTC	4				
RPS19	AGACAAGCCCTGAGAAGGCAAATGGACTGCCTA	1	GTGGGCCCCGGGGGGCAGCGGCGGGGTG	1,321	Protein coding	WT R282W
			CCCGGGGGGCAGCGGCGGGGTGCGTGGGGCGTCCGGAGTC	1,125		
			GGGGCGTCCGGAGTCCCGGGGCTGGGG	1,444		
			CCGGAGTCCCGGGGCTGGGGAGTGGGGTCGCGCAGGATCCT	0,951		
			TTGAAGGGGCCGTGGGAAGTAACGGGGGGTACC	1,303		WT
			GTGGGAAGTAACGGGGGGTACCACGGT	1,222		
			CGCTGGAGCGAAAGGATTGGGGTGGGGTCCGTGCTCTTGGCAG	0,930		
	CGGCAGGCCCGGACATGCCC	3	TTGAAGGGGCCGTGGGAAGTAACGGGGGGTACC	1,303		WT
			GTGGGCCCCGGGGGGCAGCGGCGGGGTG	1,321		WT R282W
			CCCGGGGGGCAGCGGCGGGGTGCGTGGGGCGTCCGGAGTC	1,125		
			CCGGAGTCCCGGGGCTGGGGAGTGGGGTCGCGCAGGATCCT	0,951		
			GGGGCGTCCGGAGTCCCGGGGCTGGGG	1,444		R282W
			GTGGGAAGTAACGGGGGGTACCACGGT	1,222		WT
			CGCTGGAGCGAAAGGATTGGGGTGGGGTCCGTGCTCTTGGCAG	0,930		
RPS27L	CAACAAGCCCGTTAGACCCACGGGACTTGGCA	1	GAGAACTGTTCCCCTCCCAGCCACCGCCTCTGAATT	-0,917	Protein coding	WT R282W
			CTCAGCCCTACCAGACCTCCCAGCCCACACA	-1,161		
	GGGTTTGTTTTGGGCTTCTTT		GAGAACTGTTCCCCTCCCAGCCACCGCCTCTGAATT	-0,917		
			CTCAGCCCTACCAGACCTCCCAGCCCACACA	-1,161		
	GAGCTTGCAAGAGGATCAACATGCCT		GAGAACTGTTCCCCTCCCAGCCACCGCCTCTGAATT	-0,917		
			CTCAGCCCTACCAGACCTCCCAGCCCACACA	-1,161		
	AGACAAGCCC		GAGAACTGTTCCCCTCCCAGCCACCGCCTCTGAATT	-0,917		
			CTCAGCCCTACCAGACCTCCCAGCCCACACA	-1,161		
	AAACTTTTTTTCAGAACAAAGCAAACCT		CTCAGCCCTACCAGACCTCCCAGCCCACACA	-1,161		
	AAACTTTTTTTCAGAACAAAGCAAACCT		GAGAACTGTTCCCCTCCCAGCCACCGCCTCTGAATT	-0,917		
	GGGCATGTAGTGACTTGCCC	3	GAGAACTGTTCCCCTCCCAGCCACCGCCTCTGAATT	-0,917		
	GGGCATGTAGTGACTTGCCC		CTCAGCCCTACCAGACCTCCCAGCCCACACA	-1,161		
	AGACAAGCCTGGGCA		CTCAGCCCTACCAGACCTCCCAGCCCACACA	-1,161		
	AGACAAGCCTGGGCA		GAGAACTGTTCCCCTCCCAGCCACCGCCTCTGAATT	-0,917		
SESN1	GGACAAGTCTCCACAAGTCA	2	GTCTCCCACCCACCGCAGGCAACCCCTTCCCCCGAG	-1,361	Protein coding	WT R282W

TCERG1L-AS1	AGACTTGCCTGGG CTTGTTC	4	TTCTCAGTTAGGGGCTGGACAGGGGC AGCTGCTAGA	0,917	Protein coding	WT
TEPSIN	AAACATGCAGGGA CAAGCCC	4	GGCGCGGGCCCCTTCCCCCCTTCCCAG CCCTTCCCCTCCAAGAGCCAGC	-1,327	Protein coding	WT R282W
TRIM32	CGACAAGCCCCAG CATGCTG	4	AGCATGCTGGGGAGGCGGGGCTCAGT GACGGACAGGGA	1,211	Protein coding	R282W
			TGCGCAGAGGGAGGCAGGCGGGTGGG CTGCCGG	1,030		
			GGAGCCGCGGGCGGTCAGGTAGGGGG CGGGAAGGAGGGTTGGGGACTGGGGA CCGCGGCCGGAGT	1,369		
	CAATGTGTTCATG GATAAACTTGTCT	1	GGAGCCGCGGGCGGTCAGGTAGGGGG CGGGAAGGAGGGTTGGGGACTGGGGA CCGCGGCCGGAGT	1,369		
			AGCATGCTGGGGAGGCGGGGCTCAGT GACGGACAGGGA	1,211		
			TGCGCAGAGGGAGGCAGGCGGGTGGG CTGCCGG	1,030		
TRPC2	GGACATGTCCGGG CCTGCCT	4	GGGGCCGGGCCTGGTAGAAGAGAGG	1,200	Transcribed unprocessed pseudogene	WT
			GAGGGATGAGGCAGTGCCAAGGGGGA G	1,222		
			AAAGCAGGGGAGGGCAAAGCGGGAG GATTGGTGA	1,235		
			TGGGGCCCTGGTCAGAGCGGAGGGGC	1,154		

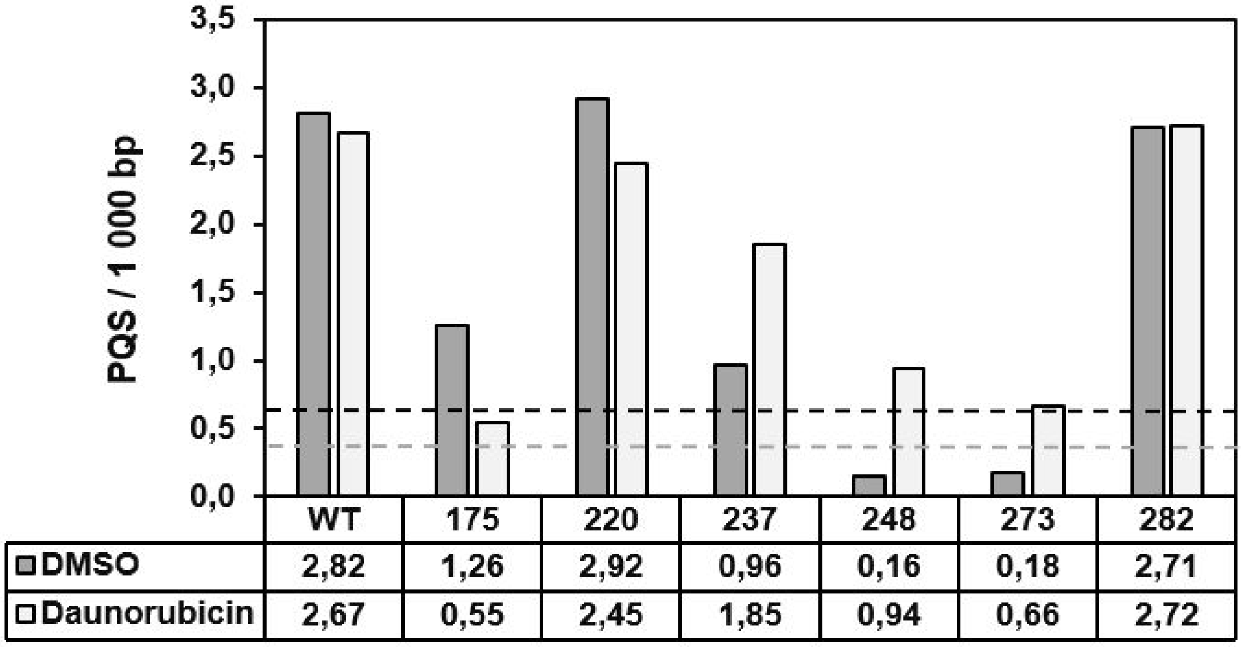

	WT	175	220	237	248	273	282
DMSO	2,82	1,26	2,92	0,96	0,16	0,18	2,71
Daunorubicin	2,67	0,55	2,45	1,85	0,94	0,66	2,72

Figure S3. Frequency of PQS in binding sites of standard and mutant p53 protein in MOLM13 cell line.

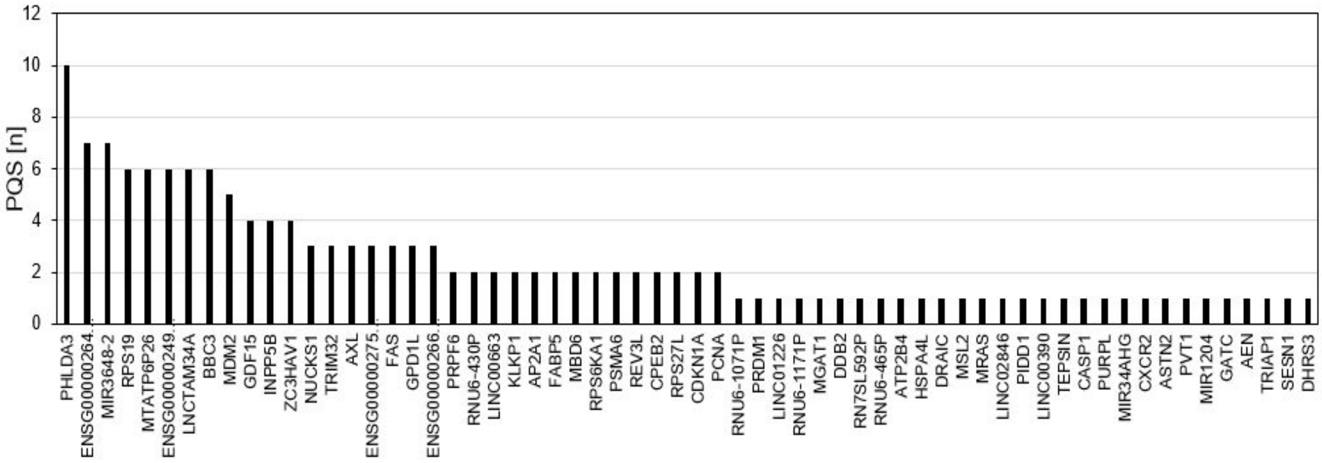

Figure S4: Representation of PQS in p53 protein binding sites in the promoter regions of individual genes in cell line MOLM13.

Table S5: Overview of genes with p53 binding sites associated with PQS in K562 and MOLM13 cell lines.

	Both	MOLM13	K562
**Genes**	*PHILDA3*	*ENSG00000249787*	*LIF-AS2*
	ENSG00000264063	*LNCTAM34A*	*NDUFAF6*
	MIR3648-2	*MTATP6P26*	*ENSG00000274868*
	RPS19	*ZC3HAV1*	*KMT2A*
	BBC3	*AXL*	*MIR6724-3*
	GDF15	*ENSG00000266100*	*TNFRSF10B*
	MDM2	*ENSG00000275693*	*TRPC2*
	REV3L	*FAS*	*PLK3*
	AEN	*GPD1L*	*ENSG00000249540*
	INPP5B	*NUCKS1*	*ENSG00000249722*
	TRIM32	*AP2A1*	*ENSG00000261135*
	CDKN1A	*FABP5*	*ENSG00000285853*
	CPEB2	*PRPF6*	*MIR3189*
	KLKP1	*PSMA6*	*MRPL27*
	LINC00663	*RNU6-430P*	*PINCR*
	MBD6	*RPS6KA1*	*TCERG1L-AS1*
	PCNA	*ATP2B4*	-
	RPS27L	*CASP1*	-
	ASTN2	*CXCR2*	-
	DDB2	*DRAIC*	-
	DHRS3	*LINC00390*	-
	GATC	*LINC01226*	-
	HSPA4L	*MIR34AHG*	-
	LINC02846	*MRAS*	-
	MGAT1	*MSL2*	-
	MIR1204	*RN7SL592P*	-
	PIDD1	*RNU6-1071P*	-
	PRDM1	*RNU6-1171P*	-
	PURPL	*RNU6-465P*	-
	PVT1	*TRIAP1*	-
	SESN1	-	-
	TEPSIN	-	-

Table S6: The p53 ChIP-ed promoters overlay with experimental datasets G4-seq analyses and BG4-chip-seq in K562 cells (https://www.balasubramanian.co.uk/research/bioinformatic-data-on-g-quadruplexes-in-genomes). A - Overlay with G4-seq analyses. B – Overlay with BG4-chip-seq in K562 cells, C – Overlay with G4-seq analyses (BBC3 and MDM2 promoters only). G4-seq experimental datasets overlay with MDM2 promoter site. D - Overlay with BG4-chip-seq in K562 cells (BBC3 and MDM2 promoters only). BG4-chip-seq datasets overlay with BBC3 promoter site.

A

Chromosome	Start P53-ChIP-seq	End P53-ChIP-seq	Start G4-seq	End G4-seq
chr19	41860640	41860672	41860667	41860772
chr19	41860640	41860672	41860667	41860772
chr19	41860651	41860677	41860667	41860772
chr19	41860651	41860677	41860667	41860772
chr19	41860686	41860728	41860667	41860772
chr19	41860686	41860728	41860667	41860772
chr12	68808868	68808893	68808672	68808972
chr12	68808868	68808893	68808672	68808972
chr17	81239686	81239734	81239625	81239715
chr6	106098754	106098799	106098773	106098878

chr8	127795667	127795712	127795663	127795768
chr8	127795667	127795712	127795663	127795768
chr8	127795667	127795712	127795663	127795768
chr8	127795667	127795712	127795663	127795768
chr8	127795667	127795712	127795663	127795768
chr1	201468970	201469010	201468931	201469081
chr1	201468990	201469016	201468931	201469081
chr1	201468997	201469021	201468931	201469081
chr1	201469010	201469038	201468931	201469081
chr1	201469019	201469044	201468931	201469081
chr1	201469023	201469048	201468931	201469081
chr1	201469026	201469057	201468931	201469081
chr1	201469041	201469065	201468931	201469081
chr1	201469077	201469124	201468931	201469081
chr4	15001411	15001457	15001442	15001517
chr4	15001437	15001463	15001442	15001517
chr12	57522377	57522416	57522387	57522492
chr12	57522377	57522416	57522387	57522492
chr12	68808758	68808803	68808672	68808972
chr12	68808758	68808803	68808672	68808972
chr12	68808781	68808809	68808672	68808972
chr12	68808781	68808809	68808672	68808972
chr9	116687364	116687428	116687352	116687787
chr9	116687364	116687428	116687352	116687787

B

Chromosome	Start P53-ChIP-seq	End P53-ChIP-seq	Start BG4-chip-seq	End BG4-chip-seq
chr20	5120061	5120096	5119958	5120144
chr20	5120061	5120096	5119958	5120144
chr20	5120143	5120167	5119958	5120144
chr20	5120143	5120167	5119958	5120144
chr19	18373974	18374008	18373918	18374053
chr19	18374020	18374054	18373918	18374053
chr19	18374032	18374060	18373918	18374053
chr19	18386065	18386107	18386029	18386234
chr1	44800094	44800118	44799771	44800448
chr1	44800094	44800118	44799771	44800448
chr1	44800099	44800129	44799771	44800448
chr1	44800099	44800129	44799771	44800448
chr19	47231216	47231240	47231142	47231479
chr19	47231220	47231310	47231142	47231479
chr19	47231303	47231327	47231142	47231479
chr19	47231335	47231374	47231142	47231479
chr15	63157423	63157453	63157379	63157557
chr15	63157423	63157453	63157379	63157557
chr15	63157423	63157453	63157379	63157557
chr15	63157423	63157453	63157379	63157557
chr15	63157423	63157453	63157379	63157557
chr15	63157423	63157453	63157379	63157557
chr15	63157423	63157453	63157379	63157557
chr9	136109741	136109787	136109538	136109762
chr19	19776535	19776560	19776399	19776719
chr19	19776535	19776560	19776399	19776719
chr1	37947053	37947077	37946986	37947140

chr1	37947053	37947077	37946986	37947140
chr19	47231220	47231310	47231142	47231479
chr12	57522377	57522416	57522305	57522438
chr12	57522377	57522416	57522305	57522438
chr9	116687238	116687275	116687209	116687330
chr9	116687238	116687275	116687209	116687330
chr9	116687318	116687350	116687209	116687330
chr9	116687318	116687350	116687209	116687330

C

Chromosome	Start P53-ChIP-seq	End P53-ChIP-seq	Start G4-seq	End G4-seq
chr12	68808868	68808893	68808672	68808972

D

Chromosome	Start P53-ChIP-seq	End P53-ChIP-seq	Start BG4-chip-seq	End BG4-chip-seq
chr19	47231303	47231327	47231142	47231479
